# A protein-encoding *CCDC7* circular RNA inhibits the progression of prostate cancer by up-regulating *FLRT3*

**DOI:** 10.1038/s41698-024-00503-2

**Published:** 2024-01-16

**Authors:** Qiong Wang, Bisheng Cheng, Sandeep Singh, Yiran Tao, Zhongqiu Xie, Fujun Qin, Xinrui Shi, Jingjing Xu, Chenxi Hu, Wanlong Tan, Hui Li, Hai Huang

**Affiliations:** 1 Department of Urology, Nanfang Hospital, Southern Medical University, Guangzhou, 510515 China; 2 Department of Pathology, School of Medicine, University of Virginia, Charlottesville, VA 22908 USA; 3 Department of Urology, Sun Yat-sen Memorial Hospital, Sun Yat-sen University, Guangzhou, 510120 China; 4 Department of Obstetrics and Gynecology, Tongji Hospital, Tongji Medical College, Huazhong University of Science and Technology, Wuhan, 430030 China; 5 Department of Urology, The Sixth Affiliated Hospital of Guangzhou Medical University, Qingyuan People’s Hospital, Qingyuan, 511518 China

**Keywords:** Tumour heterogeneity, Drug development

## Abstract

Circular RNAs (circRNAs) are a family of endogenous RNAs that have become a focus of biological research in recent years. Emerging evidence has revealed that circRNAs exert biological functions by acting as transcriptional regulators, microRNA sponges, and binding partners with RNA-binding proteins. However, few studies have identified coding circRNAs, which may lead to a hidden repertoire of proteins. In this study, we unexpectedly discovered a protein-encoding circular RNA *circCCDC7(15,16,17,18,19)* while we were searching for prostate cancer related chimeric RNAs. *circCCDC7(15,16,17,18,19)* is derived from exon 19 back spliced to exon 15 of the *CCDC7* gene. It is significantly downregulated in patients with high Gleason score. Prostate cancer patients with decreased *circCCDC7(15,16,17,18,19)* expression have a worse prognosis, while linear *CCDC7* had no such association. Overexpressed *circCCDC7(15,16,17,18,19)* inhibited prostate cancer cell migration, invasion, and viability, supporting classification of *circCCDC7(15,16,17,18,19)* as a bona fide tumor suppressor gene. We provide evidence that its tumor suppressive activity is driven by the protein it encodes, and that *circCCDC7(15,16,17,18,19)* encodes a secretory protein. Consistently, conditioned media from *circCCDC7(15,16,17,18,19)* overexpressing cells has the same tumor suppressive activity. We further demonstrate that the tumor suppressive activity of *circCCDC7(15,16,17,18,19)* is at least partially mediated by *FLRT3*, whose expression also negatively correlates with Gleason score and clinical prognosis. In conclusion, *circCCDC7(15,16,17,18,19)* functions as a tumor suppressor in prostate cancer cells through the circCCDC7-180aa secretory protein it encodes, and is a promising therapeutic peptide for prostate cancer.

## Introduction

Prostate cancer (PCa) is the most common cancer diagnosed in men in the USA, with 191,930 new diagnosed cases in 2020 which account for 21% of all cases in men^1^. Compared with other tumors, PCa is often multifocal, having topographically and morphologically distinct tumor foci due to its special anatomical structure^2^. Different tumor foci within the same patients were reported to be genetically distinct, only rarely sharing any common somatic gene mutations, including those in cancer driver genes^3^. The multifocal and heterogeneous nature of PCa are important contributors to the difficulties associated with PCa diagnosis and treatment. Gleason score has historically been the most important morphological assessment tool for localized PCa, because it includes morphological characteristics of multiple lesions^4^. Unfortunately, only the highest Gleason score of a multiple lesion is used as a criterion in clinical diagnosis, leading to inaccurate assessment and error in PCa study.

In this study, we utilized MRI imaging and pathological diagnosis to determine Gleason scores of two different tumor foci within the same patients, collected the samples, and conducted total RNA-Seq. Our initial goal was to uncover chimeric fusion RNAs that are differentially expressed in tumors with low vs. high Gleason scores. To our surprise, one transcript containing junction sequence from *CCDC7* exon19 to exon15 of the same gene was found in only low Gleason (≤3 + 4) tissues. Subsequently, we confirmed that this is not a traditional intergenic chimeric RNA, but rather a circular RNA resulted from back splicing of exon19 to exon15 of *CCDC7*.

Circular RNAs are a family of endogenous RNAs which have become a focus of biological research in recent years^5^. Studies have demonstrated that circular RNAs are involved in the progress of cell proliferation, apoptosis, metastasis, and therapy resistance in PCa^6–10^. They mainly play regulatory roles in the pathological process of PCa by acting as miRNA sponges or binding to proteins. Though a subset of circRNAs has been shown to be potentially translated into polypeptides^11–13^, this kind of phenomenon has not been reported in PCa^10^.

Here we accumulated multiple lines of evidence supporting that *circCCDC7(15,16,17,18,19)* has tumor suppressive activities: (1) it is expressed at lower levels in high-Gleason tumors than low-Gleason ones; (2) it is also expressed at lower levels in prostate cancer than in matched normal margins; (3) overexpressing *circCCDC7(15,16,17,18,19)* resulted in the suppression of prostate cancer cell viability, migration, and invasion in vitro; (4) overexpressing *circCCDC7(15,16,17,18,19)* also yielded smaller tumors in vivo; and (5) low *circCCDC7(15,16,17,18,19)* expression is correlated with worse clinical outcome. Moreover, different from the more well-known mechanisms of function, *circCCDC7(15,16,17,18,19)* shared many common characteristics with protein-encoding *circFBXW7* and *circZNF606*, and further experiments validated its protein translation activity, which is its mechanism of action for the phenotypes studied here. Additional studies found that *circCCDC7(15,16,17,18,19)* can inhibit the migration and invasion at least partially via *FLRT3*. Taken together, our study identifies a circular RNA in PCa, which may suppress the progress of PCa by encoding a protein at least partially through *FLRT3*.

## Results

### The discovery of a circular RNA in PCa

**Fig. 1:**
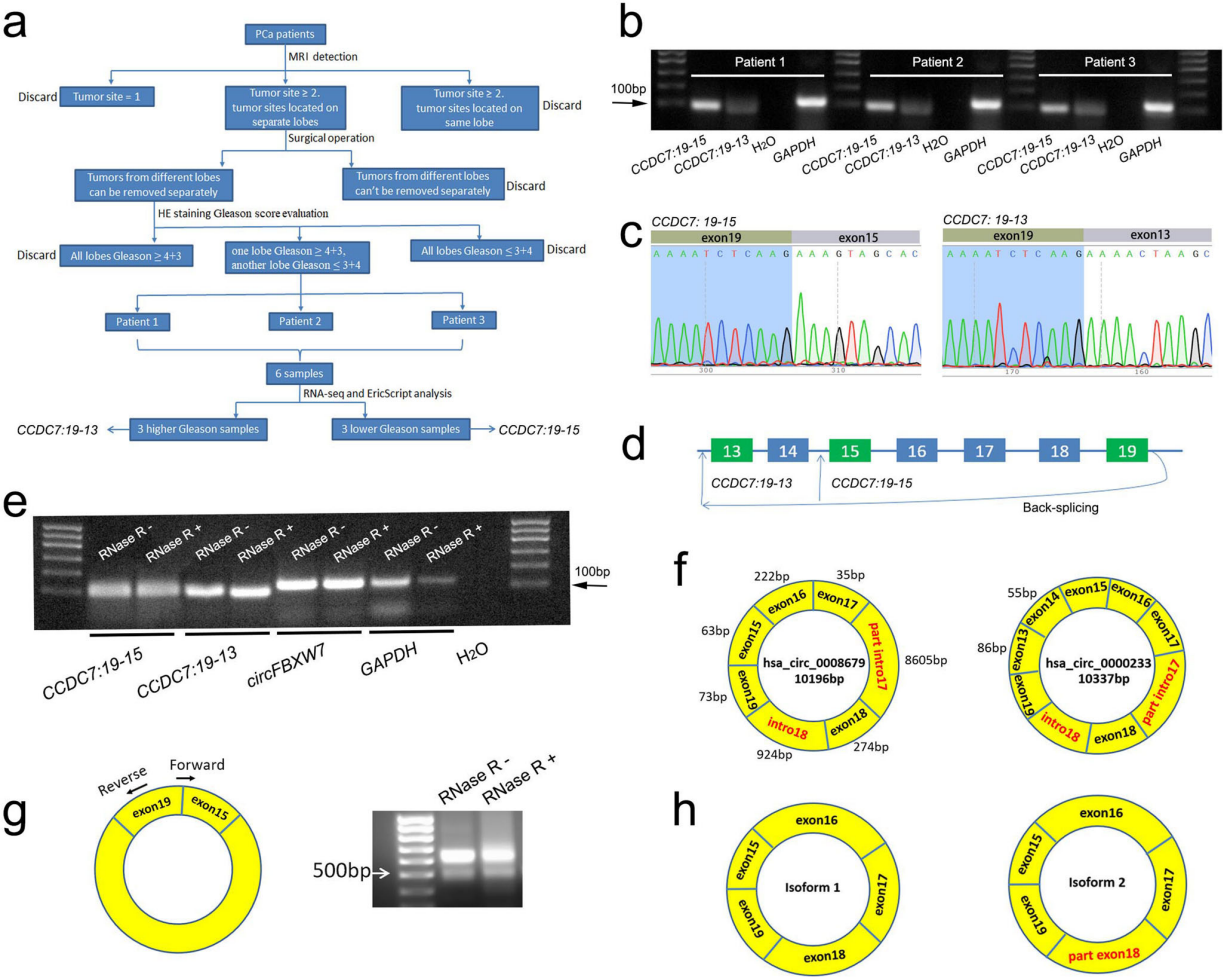
The discovery of *circCCDC7(15,16,17,18,19)* in PCa. **a** The pipeline for discovering prostate cancer chimeric RNAs: 1, based on MR reports, we focused on patients having tumor sites on two separate lobes; 2, during the surgery, the target tumors are dissected according to MR images; 3, half of the tumor was submitted to pathologist to receive a Gleason score evaluation for each tumor site; 4, selected tumors from the same patients with one lobe’s Gleason score higher than 4 + 3, and another lower than 3 + 4; 5, six tumor tissues from three patients were obtained and submitted for RNA-Seq. Finally, EricScript software was performed to predict chimeric RNAs. **b** Gel image of RT-qPCR product of *CCDC7*_*19-15*_ and *CCDC7*_*19-13*_ in three clinical patients. Mix cDNA from tumor and adjacent normal margin was used. **c** Sanger sequencing results of the validated *CCDC7*_*19-15*_ and *CCDC7*_*19-13*_. **d** The schematic diagram of *CCDC7*_*19-15*_ and *CCDC7*_*19-13*_. **e** Confirmation for *CCDC7*_*19-15*_ and *CCDC7*_*19-13*_ both being circular RNAs which *circFBXW7* was used as a control. Total mix RNA from 23 pairs PCa patients was treated with or without RNase R before performing RT-qPCR. **f** The structures of two reported circular RNAs which share the same junction sequences with *CCDC7*_*19-15*_ and *CCDC7*_*19-13*_. **g** Gel image of Touch-down PCR products of *CCDC7*_*19-15*_ using divergent primers. The complementary DNA was synthesized with random hexamer primer using total mix RNA that from 23 pairs PCa patients treated with or without RNase R. **h** The schematic diagram of different isoforms of *circCCDC7*.

Specific gene fusions and their products (fusion RNA and protein) have been widely used as cancer diagnostic markers and therapeutic targets for many years^14^. However, few recurrent fusions were found in prostate cancer with the exception of ETS family-associated gene fusions^15,16^. Recent work by our group and others has demonstrated that intergenic splicing represents an epitranscriptomic mechanism for chimeric fusion RNAs and potentially fusion proteins^17–20^. We thus hypothesized that chimeric RNAs may play important role in the malignancy of PCa and contribute to its heterogeneity. We first collected three pairs of differentially-scored PCa tissues from different lobes of the same patients according to the selection process described in the Methods. Further evaluation by three pathologists validated the Gleason scores of these six samples, which were subsequently submitted for RNA-seq (Supplementary Fig. 1). We then used the EricScript^21^ pipeline to identify recurrent chimeric RNAs. Those that specifically expressed in high Gleason score (≥4 + 3) or low Gleason score (≤3 + 4) samples were selected (Fig. 1a). To our amazement, only one transcript, an isoform of *CCDC7* with a junction site of exon19 spliced to exon15 was detected in all three low Gleason samples. In contrast, another isoform of *CCDC7* (exon19 joining to exon13) was found only in three high Gleason samples, suggesting that fusion *CCDC7*_*19-15*_ was a potential tumor suppressor gene and fusion *CCDC7*_*19-13*_ may have opposite effect. We first designed primers flanking the fusion junction site (forward primers annealing to exon19 and reverse to exon15 or 13) and used RT-qPCR to amplify these two transcripts. Agarose electrophoresis revealed bright bands for both *CCDC7*_*19-15*_ and *CCDC7*_*19-13*_ (Fig. 1b and Supplementary Fig. 2a). Further Sanger sequencing validated the existence of these two transcripts in clinical samples (Fig. 1c and Supplementary Fig. 2b). We then tested whether *CCDC7*_*19-15*_ and *CCDC7*_*19-13*_ are both circular RNAs formed by back-splicing (Fig. 1d). We used RNase R to treat the RNA before reverse transcription. The abundance of both forms did not decrease after treatment with RNase R, since GAPDH was clearly reduced, supporting that *CCDC7*_*19-15*_ and *CCDC7*_*19-13*_ are indeed circular RNAs (Fig. 1e). Interestingly, two circular RNAs with the same junction sequences are reported on circBank (http://www.circbank.cn/). Hsa_circ_0008679 (10196 bp) has the same junction to *CCDC7*_*19-15*_, and hsa_circ_0000233 (10337 bp) to *CCDC7*_*19-13*_ (Fig. 1f). In this manuscript, we focused on *CCDC7*_*19-15*_ for further study.

To test whether *CCDC7*_*19-15*_ was the reported hsa_circ_0008679, we used divergent primer pairs in which the forward primer annealing to the exon19/15 junction sequence and the reverse primer located on exon19 (Fig. 1g). To avoid the amplification of the linear form, the full length of forward primer aligns to exon19 and exon15 was 22 bp, of which 9 bp from exon19 and 13 bp from exon15. Touch-down PCR with Platinum Taq High Fidelity Kit with capability to amplify 15 kb was used to amplify the full length of *CCDC7*_*19-15*_. Interestingly, two bands between 500 bp and 700 bp were found (Fig. 1g), but not at the size of 10196 bp as in hsa_circ_0008679. Further Sanger sequencing demonstrated that the upper bright band is a circular RNA formed from exon15 to exon19 of *CCDC7* (NM_001321115.2) with a full length of 667 bp (*isoform1*; Fig. 1h and Supplementary Fig. 2c). The slightly lower band is also formed from exon15 to exon19 of *CCDC7*, but part of exon18 is lost (*isoform2*; Fig. 1h and Supplementary Fig. 2d). We suspected a cryptic splicing site on exon18 leading to the formation of the *isoform2* (Supplementary Fig. 2d)^22^. According to the recent published guidelines of naming eukaryotic circular RNAs^23^, we named the *isoform1* as *circCCDC7(15,16,17,18,19)*, and the *isoform2* as *circCCDC7(15,16,17,18S,19)*. Given that small products are favorably amplified by PCR, and the brightness of *circCCDC7(15,16,17,18S,19)* was much lower than that of *circCCDC7(15,16,17,18,19)*, *circCCDC7(15,16,17,18S,19)* must be relatively lowly expressed. We therefore decided to focus on *circCCDC7(15,16,17,18,19)*. To distinguish the isoforms, we designed another two forward primers as shown in Supplementary Fig. 3a. F1 can amplify both isoforms, whereas F2 can specifically amplify isoform1. Further agarose electrophoresis and Sanger sequencing verified the specificity of F2 (Supplementary Fig. 3b). F2 was subsequently employed for RT-qPCR in clinical samples to measure the expression level of *circCCDC7(15,16,17,18,19)*.

### *CircCCDC7(15,16,17,18,19)* is downregulated in PCa and its expression is associated with better prognosis

**Fig. 2:**
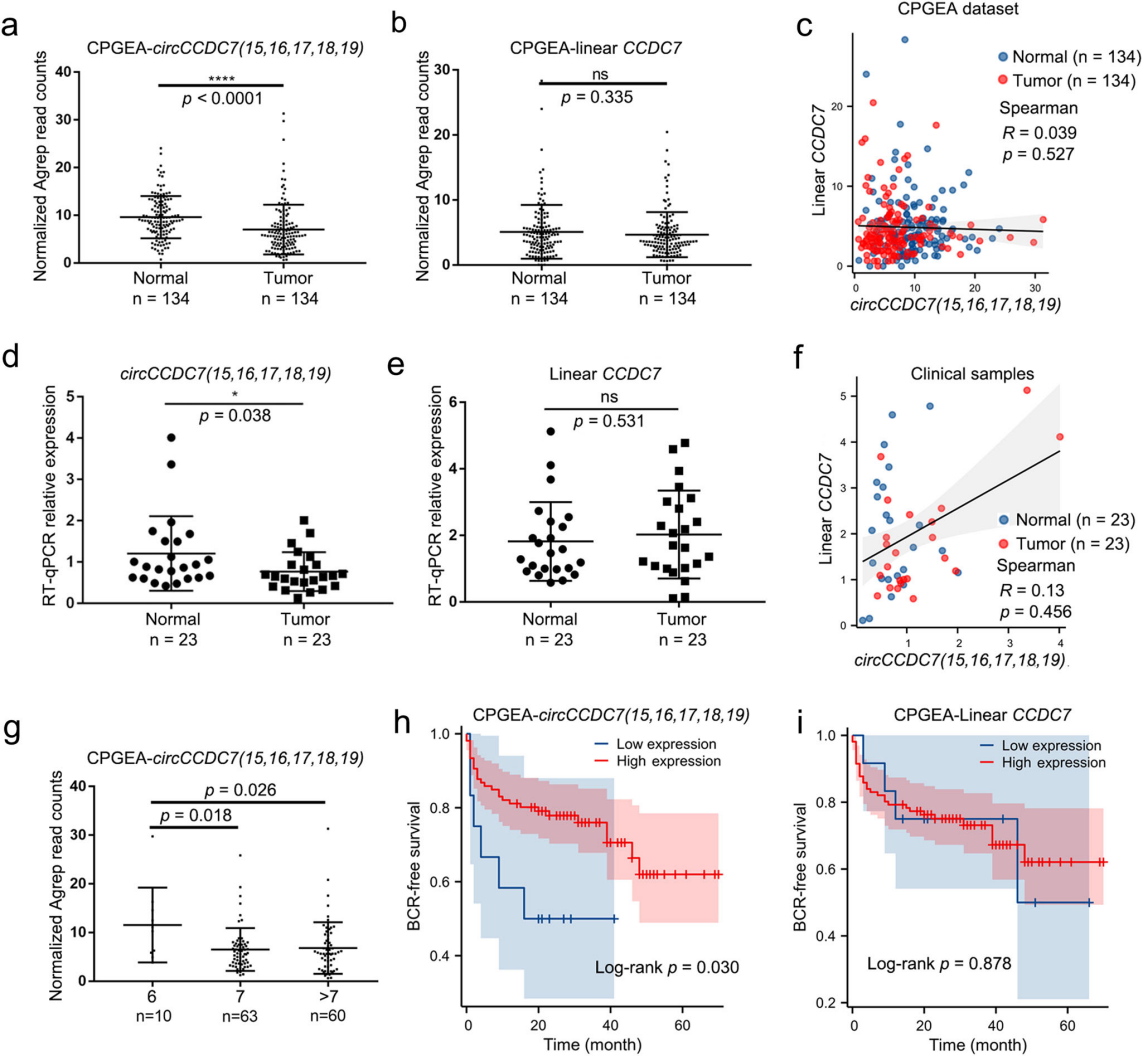
*CircCCDC7(15,16,17,18,19)* was downregulated in tumor samples and associated with good prognosis. **a**, **b** Expression of *circCCDC7(15,16,17,18,19)* (**a**) and linear *CCDC7* (**b**) in 134 pairs of PCa and normal margin samples from CPGEA by using Mann–Whitney test. **c** The absence of correlation between *circCCDC7(15,16,17,18,19)* and linear *CCDC7* in CPGEA. **d**, **e** Quantitative PCR measuring the expression of *circCCDC7(15,16,17,18,19)* (**d**) and linear *CCDC7* (**e**) in 23 pairs of clinical samples from Sun Yat-sen Memorial Hospital. The primers for linear *CCDC7* were designed to amplify the junction sequences between exon40 and exon41 to avoid all reported circular transcripts. Mann-Whitney test was used to explore the group difference. **f** The absence of correlation between *circCCDC7(15,16,17,18,19)* and linear *CCDC7* in Sun Yat-sen Memorial Hospital clinical samples. **g** Expression of *circCCDC7(15,16,17,18,19)* in different Gleason scored samples from CPGEA. Mann–Whitney test was used to explore the group difference. **h**, **i** BCR-free survival analysis of *circCCDC7(15,16,17,18,19)* (**h**) and linear *CCDC7* (**i**) in CPGEA by using normalized read counts. *p < 0.05. ****p < 0.0001.

To investigate the association of *circCCDC7(15,16,17,18,19)* with PCa, AGREP analysis was performed to quantify its expression by searching for the junction sequence in CPGEA RNA-Seq data. We first compared the expression difference between tumor and matched normal tissues and found that the *circCCDC7(15,16,17,18,19)* was expressed significantly lower in tumor samples than in the paired normal margins (Fig. 2a and Supplementary Fig. 3c). In contrast, when we used the AGREP to search for the junction sequence of *CCDC7* exon19 and exon20 (representing the linear *CCDC7*), no statistical significance was observed between tumors and normals (Fig. 2b and Supplementary Fig. 3d). The same situation was found in the TCGA database (Supplementary Fig. 3e). Consistently, we did not observe significant correlation between linear *CCDC7* expression and *circCCDC7(15,16,17,18,19)* (Fig. 2c). Experimentally, we collected 23 pairs of clinical PCa samples. RT-qPCR demonstrated that *circCCDC7(15,16,17,18,19)* was indeed expressed at lower levels in tumor samples than in the matched normal margins (Fig. 2d and Supplementary Fig. 3f). In contrast, linear *CCDC7* has no such a trend (Fig. 2e). Unsurprisingly, linear *CCDC7* expression had no statistically significant correlation with *circCCDC7(15,16,17,18,19)* in our clinical samples, either (Fig. 2f). In addition, the expression of *circCCDC7(15,16,17,18,19)* was detected at much higher levels in low Gleason samples (Gleason score = 6) in CPGEA, compared to those in the high Gleason samples (Gleason score ≥ 7) (Fig. 2g), consistent with our initial RNA-Seq data. Of note, we also found that *circCCDC7(15,16,17,18,19)* was expressed at lower levels in metastasis samples than localized samples (Supplementary Fig. 3g) in another dataset (GSE99857). These findings support that PCa is associated with reduced expression of *circCCDC7(15,16,17,18,19)*, and this effect is further increased in accordance with malignancy.

We then divided the clinical PCa cases into two groups according to the junction read counts and found that higher *circCCDC7(15,16,17,18,19)* expression predicted a better outcome (Fig. 2h), yet the linear *CCDC7* had no correlation with PCa prognosis (Fig. 2i). We also queried the TCGA prostate cancer study to replicate our findings. We could not detect the circular RNA with AGREP in TCGA prostate cancer RNA-Seq, which we presume is due to the polyA-sequencing protocol with TCGA. Additionally, we divided the expression of linear *CCDC7* into two groups according to the FPKM value in TCGA, and found that linear *CCDC7* had no statistically significant association with prostate cancer patient survival (Supplementary Fig. 3h). Univariable analysis and multivariable analysis by Cox proportional hazards modeling also demonstrated a similar prognostic value of *circCCDC7(15,16,17,18,19)* (Supplementary Table 1). Consistently, the AUC value of the ROC for tumor or normal was better in *circCCDC7(15,16,17,18,19)* (0.703) than that in linear *CCDC7* (0.534) (Supplementary Fig. 3i, j). Collectively, these results suggest that *circCCDC7(15,16,17,18,19)* is regulated differently from linear *CCDC7*, and may serve as a potential biomarker and/or prognostic marker for PCa patients.

### *CircCCDC7(15,16,17,18,19)* inhibits the viability, migration, and invasion of PCa

To investigate the effect of *circCCDC7(15,16,17,18,19)* on prostate cancer, DU145 and PC3 cell lines were infected with a lentivirus expressing the circular RNA to establish stable *circCCDC7(15,16,17,18,19)* overexpressing cell lines. We first confirmed the overexpression system through RT-qPCR and agarose electrophoresis (Supplementary Fig. 4a–c). The ΔCq in PC3 and DU145 overexpression system were about 13 and 5 respectively. To test if the overexpressed *circCCDC7(15,16,17,18,19)* levels were still in the range of natural samples, we subsequently performed RT-qPCR on prostate cell lines including PrEC LH, LNCaP, C4-2, PC3, DU145, NCI-H660 and LASCPC-01. All the cells above had a certain degree of *circCCDC7(15,16,17,18,19)* expression (Supplementary Fig. 4d), and the range of ΔCq were from 5 to 15 (Supplementary Fig. 4e). In addition, the ΔCq in the 23 pairs clinical samples ranged from 8 to 13 (Supplementary Fig. 4f). The results above suggested the overexpressed *circCCDC7(15,16,17,18,19)* levels were still in the range of natural samples.

**Fig. 3:**
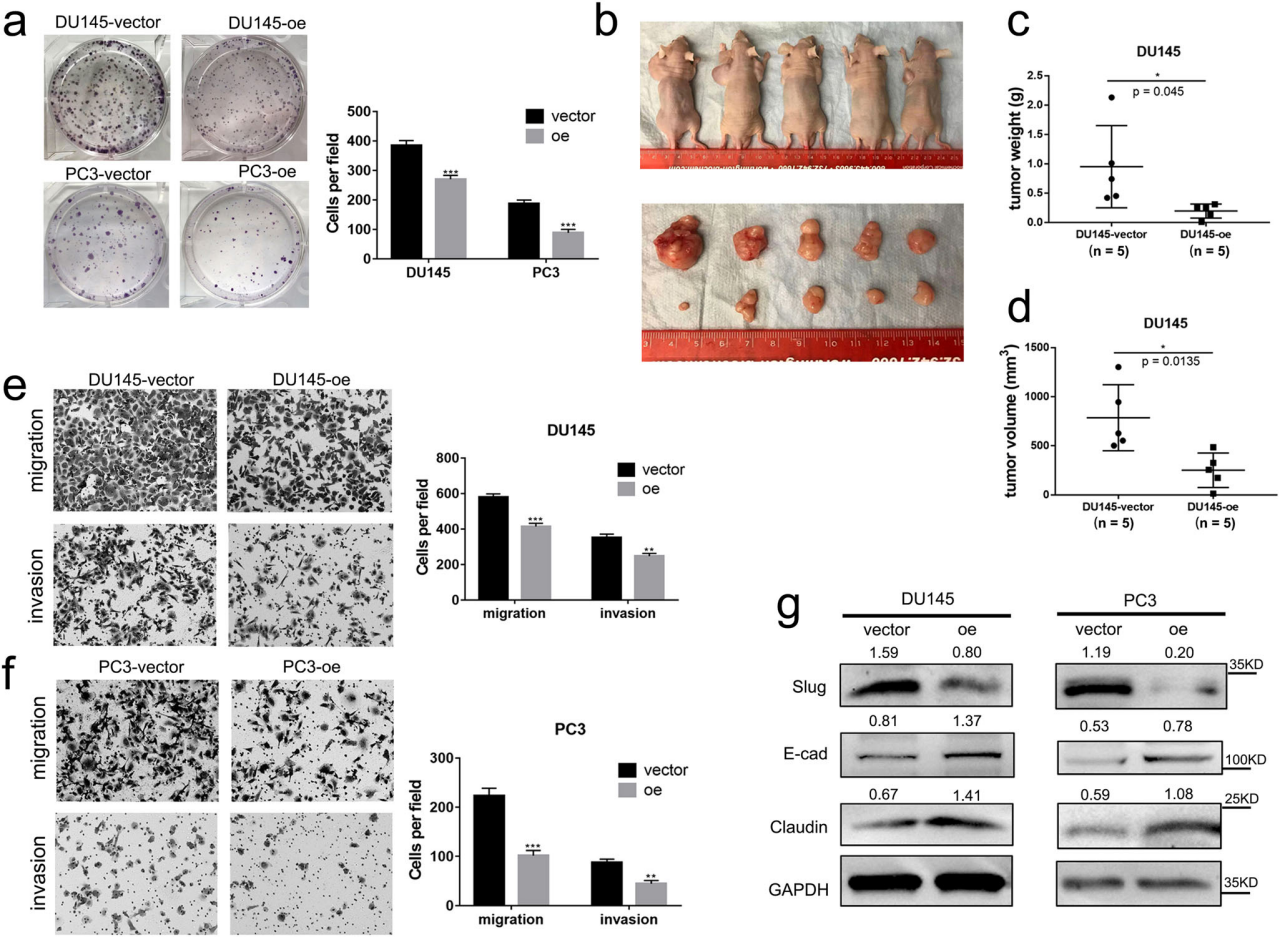
*CircCCDC7(15,16,17,18,19)* inhibits the viability, migration, and invasion of PCa. **a** Colony formation assay measuring cell viability in DU145 and PC3 cell lines (left) and histogram quantification (right). **b** Representative in vivo subcutaneous tumorigenesis images of the tumors of control group (left side in upper panel and upper side in lower panel) and *circCCDC7(15,16,17,18,19)* overexpression group (right side in upper panel and lower side in lower panel) using DU145. **c**, **d** Tumor weight and volume were measured after the tumors were surgically dissected. **e**, **f** Left panel: representative images of migration and invasion assays using DU145 (**e**) and PC3 (**f**) cells with or without *circCCDC7(15,16,17,18,19)* overexpression. Right panel: Histogram analysis of migrated and invaded cell counts. **g** Representative image of the Western blotting analysis of Slug, E-cad, and Claudin after *circCCDC7(15,16,17,18,19)* overexpression in DU145 and PC3 cells. The grayscale ratio of target protein to GAPDH was listed on top of the lanes. *p < 0.05, **p < 0.01, ***p < 0.001.

Despite that CCK8 assays did not detect the effect of *circCCDC7(15,16,17,18,19)* on cell proliferation of PC3 and DU145 (Supplementary Fig. 5a, b), colony formation assays demonstrated its inhibitory effect on cell viability (Fig. 3a). To confirm whether *circCCDC7(15,16,17,18,19)* plays an important role in tumorigenesis in vivo, we tested the effect of overexpressed *circCCDC7(15,16,17,18,19)* in a dorsal subcutaneous xenograft model. After 6 weeks, all animals in the control group had reached significant tumor volumes. In contrast, the size and weight of tumors derived from the *circCCDC7(15,16,17,18,19)* overexpression group were significantly reduced (Fig. 3b–d and Supplementary Fig. 5c). Furthermore, transwell assays showed that *circCCDC7(15,16,17,18,19)* could suppress the migration and invasion of both DU145 and PC3 cells (Fig. 3e, f). Consistently, the Epithelial-mesenchymal transition (EMT) metastasis promoting marker Slug was down-regulated, EMT suppressive genes E-cad and Claudin were up-regulated with *circCCDC7(15,16,17,18,19)* overexpression (Fig. 3g). Additionally, the androgen-dependent LNCaP cell line was also transfected with overexpression plasmid, and transwell assays showed that *circCCDC7(15,16,17,18,19)* could suppress the migration and invasion of LNCaP, suggesting that the mechanism of inhibiting PCa is not dependent on AR status (Supplementary Fig. 5d, e). Taken together, *circCCDC7(15,16,17,18,19)* inhibits the viability, migration, and invasion of PCa in vitro and in vivo.

### *CircCCDC7(15,16,17,18,19)* encodes a secreted protein

**Fig. 4:**
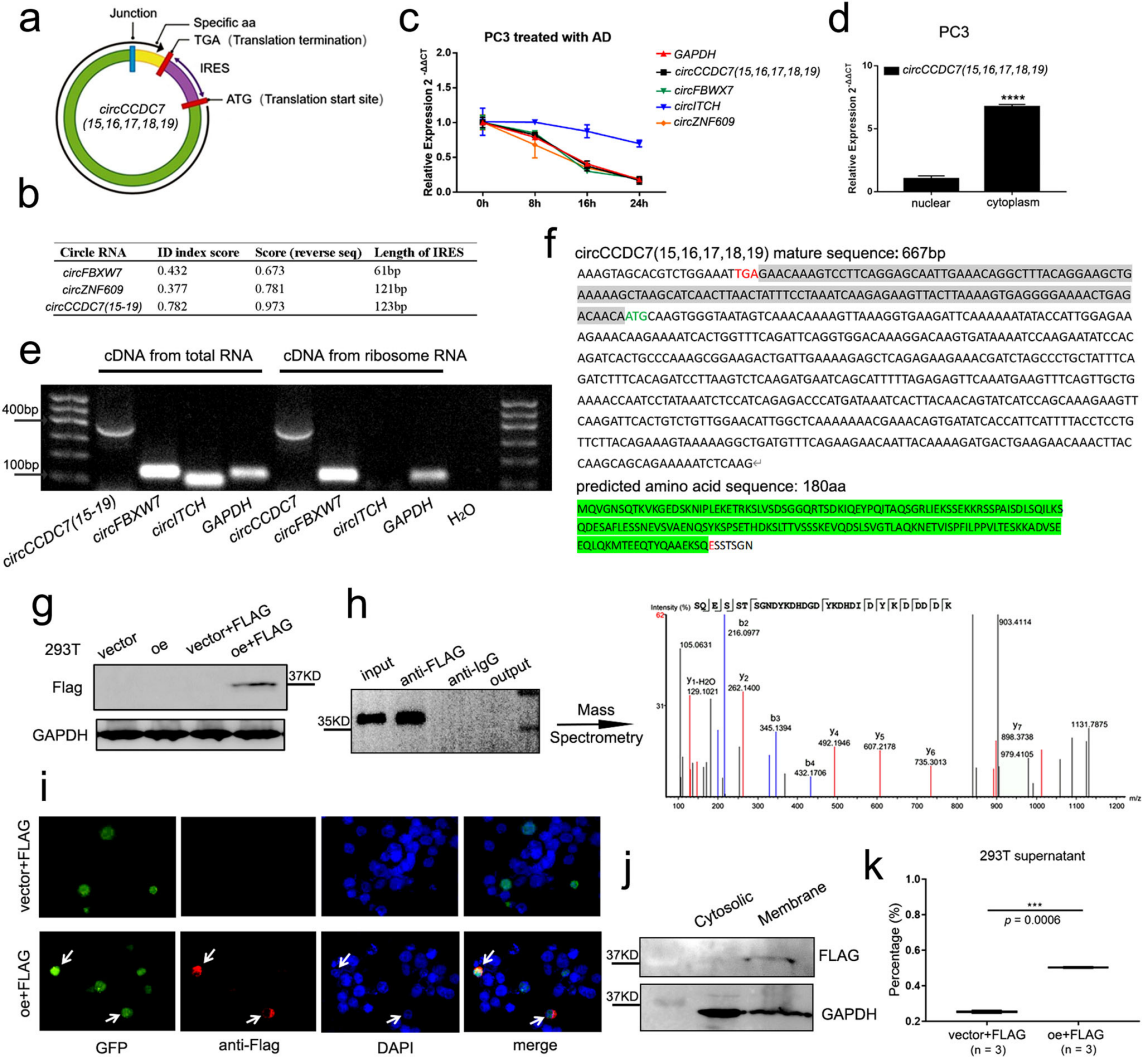
*CircCCDC7(15,16,17,18,19)* encodes a protein. **a** The putative open reading frame (ORF) in *circCCDC7(15,16,17,18,19)*. Note that the *circCCDC7(15,16,17,18,19)* junction is inside the ORF. **b** The comparison of ID index score of internal ribosomal entrance site (IRES) among *circCCDC7(15,16,17,18,19)*, *circFBXW7* and *circZNF609* using IRESfinder. **c** The relative abundance of *circFBXW7*, *circITCH, circZNF609*, *circCCDC7(15,16,17,18,19)* and *GAPDH* mRNA in PC3 cell lines detected by RT-qPCR after actinomycin D treatment at the indicated time points. The relative expression was normalized to the expression of 0 h. **d** Quantitative analysis of the expression of *circCCDC7(15,16,17,18,19)* between nuclear and cytoplasm in PC3 cells. **e** Gel image of RT-qPCR product of the *circFBXW7*, *circCCDC7(15,16,17,18,19), circITCH*, and *GAPDH* in ribosome enriched RNA. Mixed ribosome RNA from LNCaP, C4-2, PC3 and DU145 were used. **f** Upper panel: the sequence of the putative ORF is shown in black font with white background, internal start codon is shown with green font, internal stop codon is shown in red font, and the IRES sequence is shown in black word with gray background. Lower panel: the predicted amino acid sequence of circCCDC7-180aa. The amino acids with green background are the same to the linear *CCDC7* and the last seven amino acids are novel. **g** FLAG tag antibody was used to detect circCCDC7-180aa expression in 293 T cells transfected with vector or overexpression plasmid. **h** CircCCDC7-180aa was confirmed by immunoblotting after IP. The circCCDC7-180aa were extracted and subjected to mass spectrometry and junction-specific peptide (SQESSTSGN) was identified (right panel). **i** Immunofluorescence assay demonstrated that circCCDC7-180aa was mainly located around nucleus in HCT116 cell line. GFP plasmid and FLAG tagged *circCCDC7(15,16,17,18,19)* or empty vector were co-transfected. Upper is GFP+vector; Lower GFP + FLAG tagged *circCCDC7(15,16,17,18,19)*_._ **j** Western blot showed circCCDC7-180aa specifically enriched in membrane fraction. **k** ELISA assay demonstrated that circCCDC7-180aa is secreted into cell culture medium 2 days after plasmid transfection in 293 T cells. ***p < 0.001, ****p < 0.0001.

Current knowledge is that circular RNAs work mainly through the following three mechanisms: miRNA sponging, protein binding, and cap-independent translation^24^. Protein binding and miRNA sponging related circular RNAs mainly function as ncRNA (non-coding RNA). In contrast, cap-independent translation related circular RNAs mainly rely on the polypeptides/proteins they encode. We here found *circCCDC7(15,16,17,18,19)* shared many common features with protein coding circular RNAs. Firstly, after putative open reading frame (ORF) analysis, we found *circCCDC7(15,16,17,18,19)* has the basic structure for protein coding, including an internal stop codon (TGA) after junction site, an internal start codon (ATG), and the internal ribosome entry site (IRES) between the stop codon and the start codon (Fig. 4a). Secondly, after IRES analysis (https://github.com/xiaofengsong/IRESfinder), we found the ID index score of IRES in *circCCDC7(15,16,17,18,19)* was much higher than that in other protein coding circular RNAs such as *circFBXW7* or *circZNF609*^25,26^, predicts its higher binding potential to ribosomes (Fig. 4b). Thirdly, circular RNAs tend to be more stable. However, an actinomycin D assay showed that the half-life of the *circCCDC7(15,16,17,18,19)* was about 12 h, which is similar to that of protein-coding RNAs: *GAPDH*, *circFBXW7* and *circZNF609*, while the half-life of a traditional circular RNA *circITCH* working as the sponge of miRNA^27,28^ exceeded 24 h (Fig. 4c). Furthermore, many ncRNAs tend to reside more in the nucleus regulating transcription. However, nucleo-cytoplasmic separation showed that *circCCDC7(15,16,17,18,19)*, *circFBXW7* and *circZNF609* were all enriched in cytoplasm (Fig. 4d and Supplementary Fig. 6a, b), consistent with their protein-coding roles. Lastly, since RNA-ribosome complex is necessary for protein translation, we extracted mixed ribosomes from cultured PCa cells (LNCaP, C4-2, PC3 and DU145) and validated the existence of *circCCDC7(15,16,17,18,19)*, linear mRNA *GAPDH* and *circFBXW7* in the complex, yet the traditional *circITCH* was not detected (Fig. 4e). Taken together, these results supported that *circCCDC7(15,16,17,18,19)* was a protein-coding circular RNA.

Based on ORF analysis, we predicted a 180aa protein (circCCDC7-180aa) encoded by *circCCDC7(15,16,17,18,19)* (Fig. 4f). 173aa out of 180aa are the same to linear CCDC7 protein, while the last seven amino acids are uniquely encoded by exon19 looping back to exon15. We further used the specific junction amino acids (SQESSTSGN) BLASTP against UniProtKB/Swiss-Prot database (https://www.uniprot.org) and found no exact match (Supplementary Fig. 6c, d), suggesting that this peptide cannot be derived from a linear transcript of another gene.

To further confirm the protein-coding potential of *circCCDC7(15,16,17,18,19)*, we inserted 3 × FLAG just before the stop codon and the full-length sequences were cloned to plenti-ciR-GFP-T2A vector plasmid (Supplementary Fig. 7a). After transient transfection in 293 T cells, we detected a clear band around 37kd (Fig. 4g). We could also detect the same bands in PC3 and DU145 despite their relatively lower transfection efficiency (Supplementary Fig. 7b). Importantly, circCCDC7-180aa was further confirmed by immunoblotting after IP and mass spectrometry, which identified the junction-specific peptides (SQESSTSGN) (Fig. 4h). Immunofluorescence was conducted to visualize the expression of the protein. We chose to use HCT116 cells because its higher transfection efficiency and cell shape. The results showed that circCCDC7-180aa was mainly located around nucleus resembling Golgi localization (Fig. 4i). Further immunofluorescence also confirmed that circCCDC7-180aa mainly positioned around the nucleus in the stable overexpression DU145 (Supplementary Fig. 7c). We thus suspected that circCCDC7-180aa is processed in Golgi and subsequently transferred to cell membrane. To this end, we further isolated membrane from cytosolic fraction, and used Western blot to demonstrate that circCCDC7-180aa was indeed enriched in membrane fraction (Fig. 4j). Because of the localization to Golgi, we further tested whether the protein is also secreted. We first performed an ELISA assay on cell culture supernatant, and the results verified our conjecture (Fig. 4k). Secondly, we harvested the supernatant, and after lyophilization, we detected the correct sized band on a Western blot (Supplementary Fig. 7d). The following mass spectrometry also identified the peptides of circCCDC7-180aa (Supplementary Fig. 7e, f). In summary, we found evidence that *circCCDC7(15,16,17,18,19)* encodes a protein with partial novel sequence, which is readily found in cell culture supernatant, suggesting that it is secreted.

### *CircCCDC7(15,16,17,18,19)* suppresses the progression of PCa by its encoded protein

**Fig. 5:**
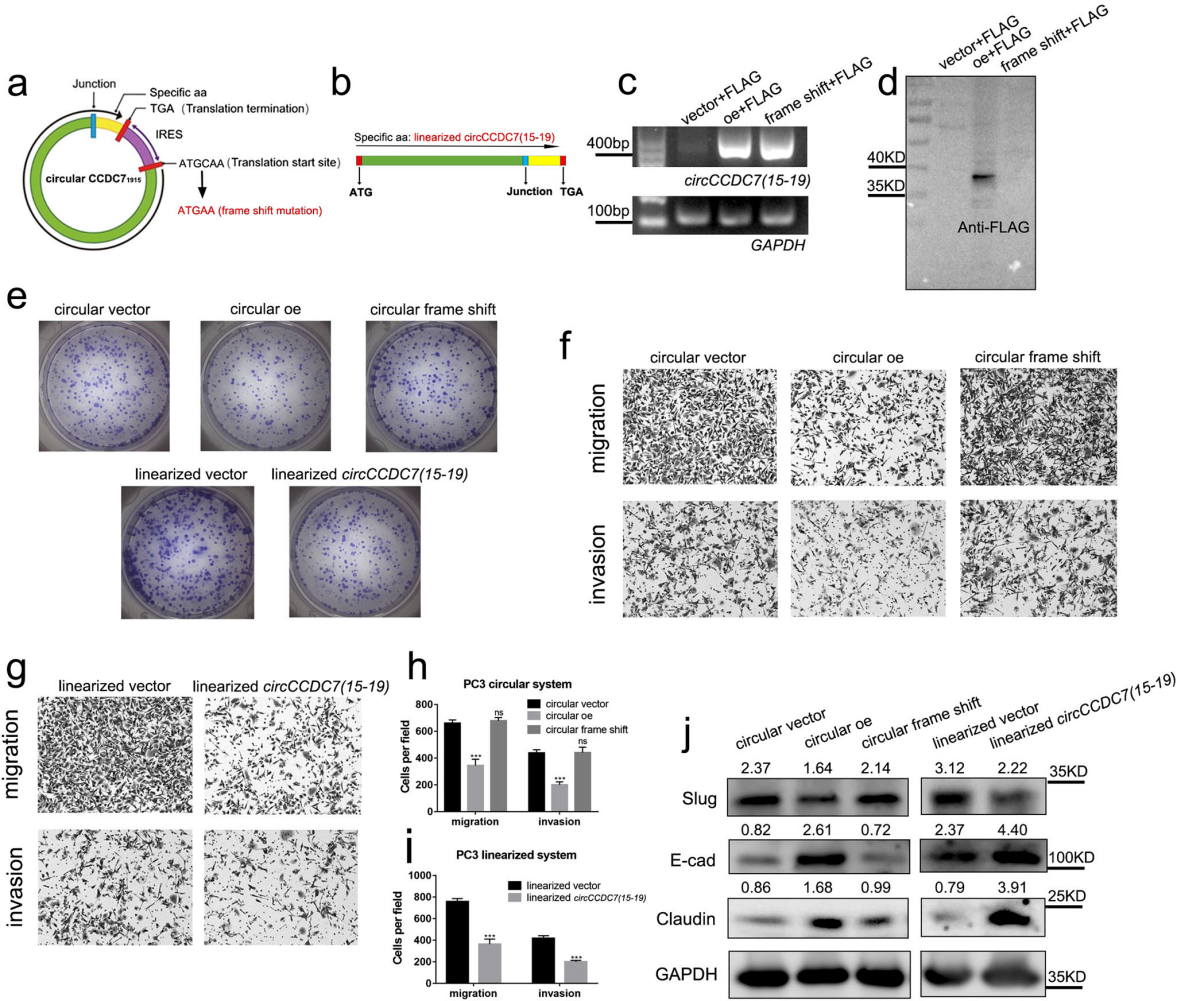
*CircCCDC7(15,16,17,18,19)* suppresses the progression of PCa by its encoded protein. **a** Schematic diagram of *circCCDC7(15,16,17,18,19)* frame shift mutant which one base after the start codon (ATG) was deleted. **b** Schematic diagram of linearized *circCCDC7(15,16,17,18,19)* ORF plasmid. **c** Gel image of RT-qPCR product of *circCCDC7(15,16,17,18,19)* from 293 T cells transfected with vector, *circCCDC7(15,16,17,18,19)* overexpression or frame shift mutation group. **d** Western blot demonstrated the absence of FLAG tagged protein with the frame shift mutant. **e** Colony formation assay measuring cell viability in PC3 cells when different plasmids were transfected. **f**, **g** Representative images of migration and invasion assays using PC3 after different plasmids transfection. **h**, **i** Histogram analysis of migrated and invaded cell counts. **j** Representative image of the Western blotting analysis of Slug, E-cad, and Claudin protein levels after transfection with different plasmids in PC3 cells. The grayscale ratio of target protein to GAPDH was listed on top of the lanes. ***p < 0.001.

Although some protein-coding circular RNAs have been discovered in recent years, such as *circFBXW7*^25^ and *circZNF609*^26^, some other studies have demonstrated that they can also exert their functions by acting as miRNA sponges^9,29^. Therefore, we felt the need to determine the specific mechanism by which *circCCDC7(15,16,17,18,19)* affects the progression of PCa. To distinguish the potential role of as a ncRNA vs. protein coding, we mutated the plasmid of *circCCDC7(15,16,17,18,19)* by deleting one base after the start codon (frame shift mutation) (Fig. 5a and Supplementary Fig. 7g), which presumably will not affect the putative ncRNA activity. As a control, we cloned the same ORF plus 3 × FLAG tag into a linear vector (Fig. 5b and Supplementary Fig. 7h). Further RT-qPCR and agarose electrophoresis validated that *circCCDC7(15,16,17,18,19)* and mutated *circCCDC7(15,16,17,18,19)* was significantly overexpressed in both wild-type and frame shift mutant groups (Fig. 5c), however, we could not detect the signal of FLAG in frame-shifted *circCCDC7(15,16,17,18,19)* via Western blot (Fig. 5d). We transfected these plasmids into PC3 and DU145 cells and measured their effect on cell viability, migration, and invasion. The frame shift mutant *circCCDC7(15,16,17,18,19)* did not affect the viability of PCa, while both the wild-type *circCCDC7(15,16,17,18,19)* and the linear construct decreased the viability significantly (Fig. 5e and Supplementary Fig. 8a). Similarity, transwell assay demonstrated *circCCDC7(15,16,17,18,19)* with frame shift mutation lost its inhibitory function on migration and invasion of PCa (Fig. 5f, h; Supplementary Fig. 8b, d). In contrast, both the wild-type *circCCDC7(15,16,17,18,19)* and the linear construct decreased the ability of migration, and invasion in PC3 and DU145 (Fig. 5g, i; Supplementary Fig. 8c, e). The metastasis related markers also showed a consistent trend (Fig. 5J and Supplementary Fig. 8f). These results support that the tumor suppressive ability is due to the protein *circCCDC7(15,16,17,18,19)* encodes, but not by a ncRNA-mediated mechanism.

**Fig. 6:**
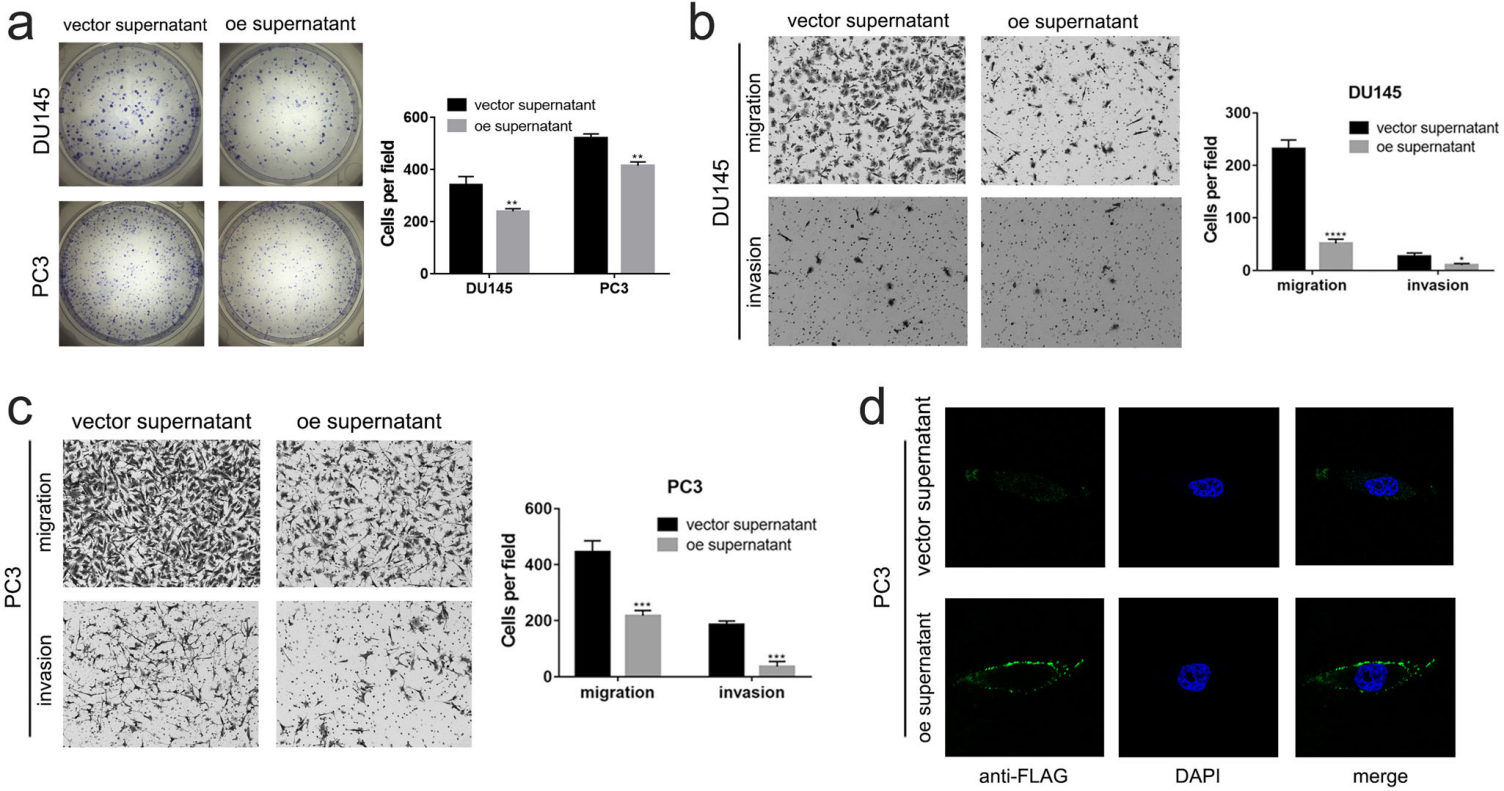
*CircCCDC7(15,16,17,18,19)* encodes a secretory protein that suppresses PCa. **a** Colony formation assay measuring cell viability in PC3 and DU145 cells after supernatant from vector or *circCCDC7(15,16,17,18,19)* overexpression plasmid transfected 293 T treatment. Supernatant to media ration, 1:1. **b**, **c** Representative images of migration and invasion assays of DU145 (**b**) and PC3 (**c**) with vector or oe supernatant treatment. Supernatant to media ration, 1:1. **d** Representative images of immunofluorescence of PC3 with enriched supernatant treatment which from vector or *circCCDC7(15,16,17,18,19)* overexpression plasmid transfected 293 T. *p < 0.05, **p < 0.01, **p < 0.001.

Since we demonstrated that the *circCCDC7(15,16,17,18,19)* encoded protein can be secreted outside of the cells, we collected cell media from 293 T cells transfected with OE construct of *circCCDC7(15,16,17,18,19)*_._ We then added these media to cultured PC3 and DU145. Impressively, a 1:1 ratio of supernatant from *circCCDC7(15,16,17,18,19)* OE 293 T cells to cell culture media on both PCa cell lines lead to obviously reduced cell viability (Fig. 6a) Similarly, reduced migration and invasion were also observed (Fig. 6b, c), supporting a potential therapeutic potential for the secreted protein. To visualize the location of the circCCDC7-180aa on recipient cells, the enriched circCCDC7-180aa media from cell media of 293 T transfected with FLAG tagged *circCCDC7(15,16,17,18,19)* was added to the adherent wide-type PC3 cells. Immunofluorescence confirmed that this protein was mainly enriched on the cell membrane, suggesting that circCCDC7-180aa may regulate the progression of PCa via some receptor-ligand interaction (Fig. 6d).

### FLRT3 is a downstream target of *circCCDC7(15,16,17,18,19)*

**Fig. 7:**
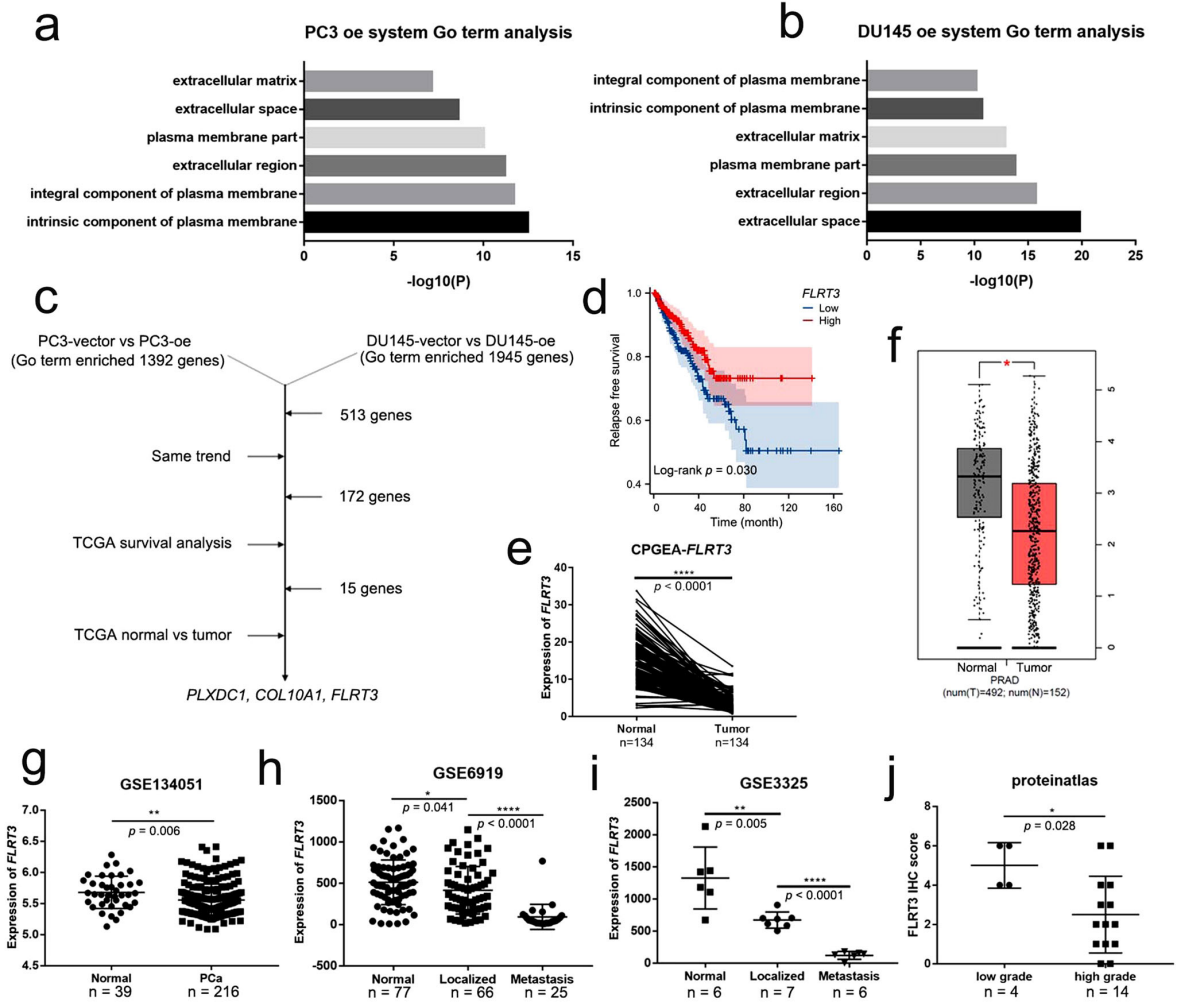
*FLRT3* is a potential downstream of circCCDC7-180aa. **a**, **b** Gene ontology analyses of differentially expressed genes after *circCCDC7(15,16,17,18,19)* overexpression in PC3 (**a**) and DU145 (**b**) cell lines. **c** Three downstream genes selected from the RNA-seq were examined with TCGA database and TCGA relapse-free survival analyses. **d** Relapse-free survival analysis of *FLRT3* in TCGA. **e** Expression of *FLRT3* in 134 pairs of PCa and normal margin samples from CPGEA. **f**, **g** Expression of *FLRT3* in PCa and normal margin samples from TCGA (**f**) and GSE134051 (**g**). **h**, **i** Expression of *FLRT3* in normal, localized and metastasis samples from GSE6919 (**h**) and GSE3325 (**i**). **j** Expression of FLRT3 protein in PCa samples of different grades from Proteinatlas. *p < 0.05, **p < 0.01, ****p < 0.0001.

To investigate downstream functional mechanism of *circCCDC7(15,16,17,18,19)*, next-generation RNA sequencing was performed on PC3 and DU145 cells with *circCCDC7(15,16,17,18,19)* overexpression. Genes with log_2_ |Fold Change | ≥1 were considered significantly differentially expressed. Consistent with its role in migration and invasion, we found enriched cellular components important for metastasis, such as extracellular matrix and extracellular region by gene ontology term analysis (Fig. 7a, b). There are 1,392 GO term enriched genes in PC3 and 1,945 genes in DU145, of which there were 513 overlaps. Among them, 172 genes with the same changing trend in both cell lines were chosen for further analysis. After discarding genes with no association with PCa prognosis, and those with no significant expression difference between tumor and normal tissues in TCGA, three genes remained (Fig. 7c). Among them, *PLXDC1* and *COL10A1* are both up-regulated after *circCCDC7(15,16,17,18,19)* overexpression, but correlated with worse prognosis (Supplementary Fig. 9a, b). In contrast, high expression of fibronectin leucine rich transmembrane protein 3 (*FLRT3*) (log_2_ |Fold Change | = 1.5 and 3.7, respectively) is correlated with a good prognosis (Fig. 7d). Thus, we decided to focus on *FLRT3* as it is most likely to be a downstream mediator of the tumor suppressive activity of *circCCDC7(15,16,17,18,19)*. Further RT-qPCR and Western blot also validated that *circCCDC7(15,16,17,18,19)* indeed upregulates the expression of *FLRT3* at RNA and protein level (Supplementary Fig. 9c–e). Consistently, we collected enriched cell media from 293 T cells transfected with OE construct of *circCCDC7(15,16,17,18,19)*_._ We then added these media to cultured PC3 and DU145 and found up-regulation of *FLRT3* (Supplementary Fig. 9f, g). Lastly, we observe positive correlation between *FLRT3* expression and *circCCDC7(15,16,17,18,19)* in CPGEA dataset (R = 0.411, *p* < 0.001) (Supplementary Fig. 9h).

To explore the role of *FLRT3* in PCa, we then compared the RNA level of *FLRT3* between normal and tumor samples in CPGEA and TCGA. Similar to *circCCDC7(15,16,17,18,19)*, *FLRT3* was significantly downregulated in tumor samples (Fig. 7e, f). Analysis with another dataset GSE134051 showed a similar result (Fig. 7g). Importantly, higher mRNA levels of *FLRT3* were associated with lower Gleason scores, which is similar to our observation with *circCCDC7(15,16,17,18,19)* (Supplementary Table 2). Since there were few cases of distant metastasis in TCGA and CPGEA, other GEO datasets GSE6919 and GSE3325 were employed to examine the relationship between *FLRT3* and tumor metastasis. As expected, the expression of *FLRT3* was the lowest in metastases, when compared with localized and normal samples (Fig. 7h, i). In addition to mRNA level comparisons, we further analyzed FLRT3 protein level in The Human Protein Atlas (https://www.proteinatlas.org/), which contains the IHC pictures from 18 patients with different grades (Supplementary Fig. 9i). The immunoreactivity score was calculated as the intensity score multiplied by the quantity score (Supplementary Table 3), and the results showed that at protein level, higher level of FLRT3 was correlated with lower PCa grades (Fig. 7j). Taken together, we hypothesized *circCCDC7(15,16,17,18,19)* regulates the progress of PCa through *FLRT3*.

### Knocking down *FLRT3* rescues the effect of *circCCDC7(15,16,17,18,19)* on PCa

**Fig. 8:**
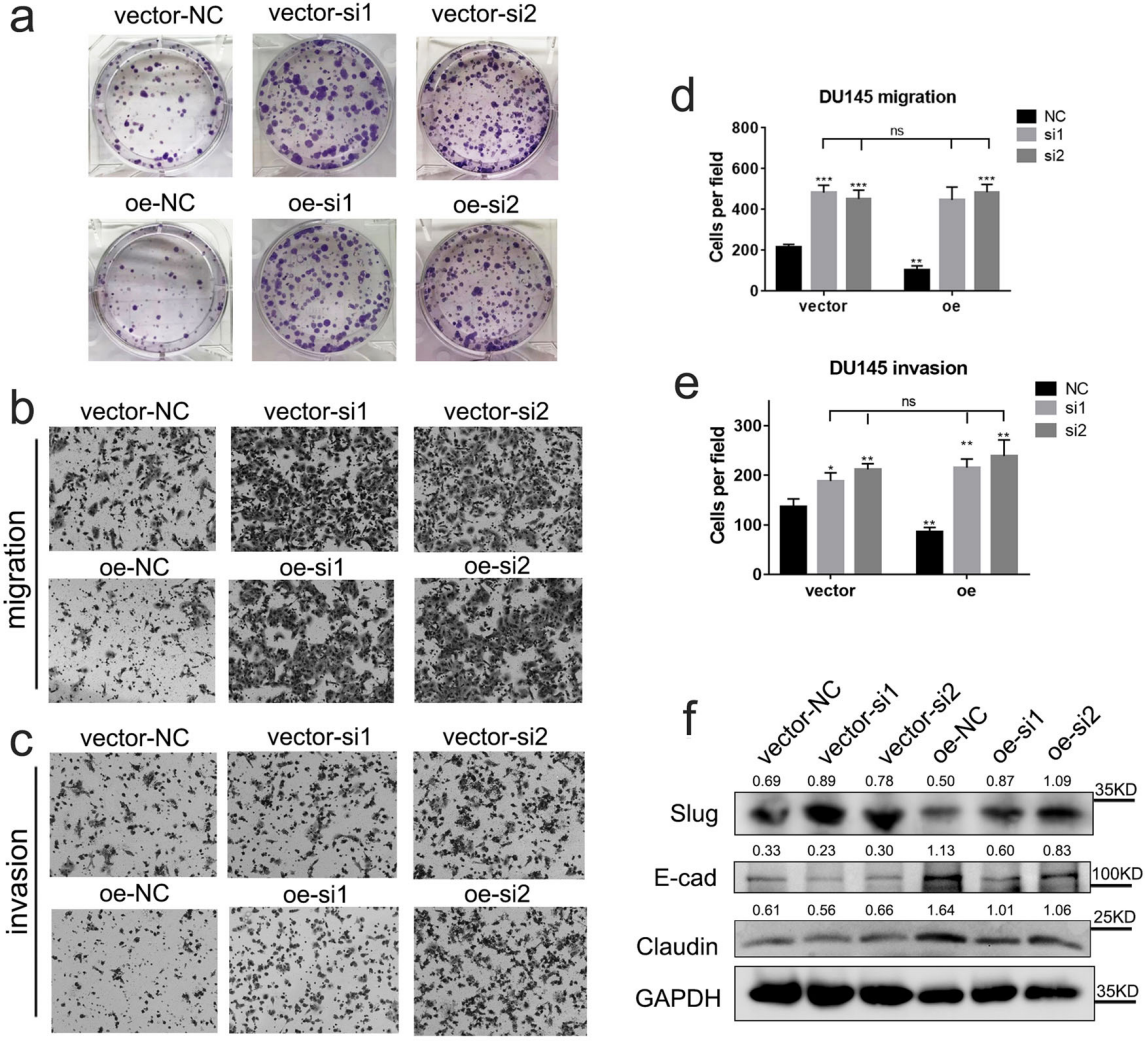
Knocking down *FLRT3* offsets the effect of *circCCDC7(15,16,17,18,19)* on PCa. **a** Colony formation assay measuring cell viability with DU145 *circCCDC7(15,16,17,18,19)* overexpressing (oe) or control cells (vector) combined with *FLRT3* knocking down (si1 and si2) or control siRNA (NC). **b**, **c** Representative images of migration and invasion assays using DU145 *circCCDC7(15,16,17,18,19)* oe combined with *FLRT3* knocking down. **d**, **e** Histogram analysis of migrated and invaded cell counts. **f** Representative image of the Western blotting analysis of Slug, E-cad, and Claudin protein levels using DU145 *circCCDC7(15,16,17,18,19)* oe combined with *FLRT3* knocking down. The grayscale ratio of target protein to GAPDH was listed on top of the lanes. *p < 0.05, **p < 0.01, ***p < 0.001.

*FLRT3* encodes a member of the fibronectin leucine rich transmembrane protein (FLRT) family, which may function in cell adhesion and/or receptor signaling^30^. *FLRT3* is considered a ligand of neuronal receptor latrophilin 1 (*LPHN1*) and has been reported to be involved in the immune escape of breast cancer cells of different origins^31^. However, there has been no reports regarding its role in prostate cancer. We overexpressed *circCCDC7(15,16,17,18,19)* and then used siRNAs to knockdown the expression of *FLRT3* in PCa cells. RT-qPCR and Western blot validated the knocking down efficiency in vector and *circCCDC7(15,16,17,18,19)* overexpression groups (Supplementary Fig. 10). Interestingly, reducing the level of *FLRT3* could cancel out the suppressive effect caused by *circCCDC7(15,16,17,18,19)* on the viability of PCa cells (Fig. 8a). Similarity, the reduced cell migration/invasion caused by *circCCDC7(15,16,17,18,19)* can be entirely offset by knocking down *FLRT3* (Fig. 8b–e), and a similar change was found in metastasis related markers Slug, E-cad and Claudin (Fig. 8f), suggesting that *FLRT3* functions downstream of *circCCDC7(15,16,17,18,19)*. All these results indicate that *circCCDC7(15,16,17,18,19)* inhibits metastasis and viability of PCa cell at least partially in a *FLRT3*-dependent manner (Supplementary Fig. 11).

## Discussion

Compared with other tumors, prostate cancer has high heterogeneity, which poses obstacles for its diagnosis and treatment. This high intratumoral heterogeneity of PCa may manifest in many aspects, including genomics, epigenetics, and phenotype^32^. Gleason score has long been regarded as the most important evaluation of PCa and is significantly correlated with the outcomes of patients^33^. We here used a series of screening criteria, and finally chose tumor samples from different lobes with different Gleason scores from the same patients. We believe that in depth analysis of these samples will enhance our understanding of the heterogeneity of prostate cancer.

In this study, we found *circCCDC7(15,16,17,18,19)* specifically expressed in low Gleason samples. It should be mentioned that using AGREP, we failed to detect *circCCDC7(15,16,17,18,19)* in TCGA dataset possibly due to its polyA sequencing platform. However, we successfully found reads supporting its existence in other datasets where the total RNA sequencing was performed, suggesting that total RNA sequencing is necessary to discover this circular RNA. Additionally, the circRNA sequences in public database were often only predicted and sometimes contain introns. Thus, it is possible that *circCCDC7(15,16,17,18,19)* we found here is the same circular RNA has been discovered before as hsa_circ_0008679.

Circular RNAs have been promoted as cancer diagnostic markers and therapeutic targets^34,35^, and interest in the machinery that drives their genesis and function has intensified over the last few years. Until now, most studies demonstrated that circular RNAs exert their regulatory functions through miRNA sponging or protein binding^36^. Here, we accumulated multiple lines of evidence supporting that *circCCDC7(15,16,17,18,19)* encodes a protein, which is the mechanism of action for its tumor suppressive activity: (1) by analyzing its sequence, we found a strong IRES sequence between the stop and start codon on the circle; (2) in contrast to other circular RNAs that are more stable, *circCCDC7(15,16,17,18,19)* has a relatively short half-life similar to other protein-coding circRNAs; (3) *circCCDC7(15,16,17,18,19)* mostly resides in cytosolic fraction, consistent with its protein coding role, not the usual ncRNA role in regulating transcription in nucleus; (4) *circCCDC7(15,16,17,18,19)* is enriched in ribosomal-enriched RNA fractions; (5) circular RNA expression constructs with in frame FLAG resulted in protein expression confirmed by Western blot and mass spectrometry analysis; and (6) conversely, a frame shift mutant with only one base deletion at the start codon failed to function, whereas a linear ORF expression construct behaved the same as *circCCDC7(15,16,17,18,19)*_._ Of note, a given circular RNA may work through many different mechanisms. For example, *circFBXW7* attenuates malignant progression in lung adenocarcinoma by sponging miR-942-5p, as well as encoding FBXW7-185aa to repress glioma tumorigenesis^25,37^. Therefore, it will not be surprising if *circCCDC7(15,16,17,18,19)* exerts its function through different mechanisms in different situations.

It is also interesting to notice that among the few examples of circRNAs which encode proteins, most have anti-tumor effects: *circLINC-PINT* has been reported to produce PINT87aa that suppresses glioblastoma^38^; FBXW7-185aa produced from *circFBXW7* mentioned above is known to inhibit cancer proliferation and migration^39^; circPPP1R12A-73aa from *circPPP1R12A* was found to display a tumor suppressive role in colon cancer^40^. However, most of them cannot be secreted, so the possibility of being a therapeutic agent is greatly reduced. Just like insulin for diabetes, circCCDC7-180aa encoded by *circCCDC7(15,16,17,18,19)* supports a new direction for PCa intervention.

Although we have confirmed that *FLRT3* is a potential downstream mediator, we did not explore the specific mechanism how *circCCDC7(15,16,17,18,19)* regulates *FLRT3*. *FLRT3* is a putative type I transmembrane protein containing 10 leucine-rich repeats, a fibronectin type III domain, and an intracellular tail^41^, which is supposed to regulate neuronal cell outgrowth and morphogenesis^42^. Some other studies also found that *FLRT3* plays an important role in other biological processes. For example, *FLRT3* is reported to be involved in the prevention of anti-tumor immunity via Tim-3-Galectin-9 pathway^31^. Jauhiainen et al., demonstrated *FLRT3* has a role in endothelial cells via regulation of VEGF-stimulated EC-survival, migration, and tube formation^43^. Ma et al., showed *FLRT3* is a significant and independent prognostic signature for lung squamous cell carcinoma^44^. However, there has been no study related to prostate cancer. We did not detect by immunoprecipitation its interaction with circCCDC7-180aa and (data not shown), suggesting that *FLRT3* may be indirectly regulated by *circCCDC7(15,16,17,18,19)*. It is possible that circCCDC7-180aa acts as a ligand after its extracellular secretion, and then receptor-ligand complexes formed to regulate the expression of *FLRT3*. Additionally, we believe that the downstream targets of *circCCDC7(15,16,17,18,19)* are not just *FLRT3*. More studies in PCa have revealed the critical role of the tumor microenvironment in the initiation and progression to advanced disease^45^, in which secreted protein is one of the important tools for communicating between tumor and its microenvironment^46^. Therefore, our future research will be committed to the influence of circCCDC7-180aa on other cells in tumor microenvironment, such as tumor-related macrophages (TAMs), cancer-associated fibroblasts (CAFs), and even T cells.

## Methods

### Clinical samples

Clinical samples were selected and processed as follows: Patients with more than two tumor sites which distributed in at least two separate lobes were selected based on MR reports, and target tumors were dissected according to MR images. Half of each tumor was submitted to the Pathology department to receive a Gleason score evaluation. Three patients with variable Gleason scores, defined as one lobe’s Gleason score higher than 4 + 3, and another lower than 3 + 4, were selected for further study. We chose a Gleason score of 7 as the cut-off point because the patients with GS < 7 (ISUP grade 1) were considered to be low-risk, GS = 7 (ISUP grade 2/3) were considered as intermediate-risk, and GS > 7 (ISUP grade 4/5) were considered to be high-risk according to internationally recognized EAU guidelines for prostate cancer (https://uroweb.org/guidelines/prostate-cancer). A total of six tumor tissues from three patients were obtained, and submitted for RNA-Seq (Supplementary Fig. 1).

Another set of fresh tumor and adjacent normal tissues of 23 PCa patients from Sun Yat-sen Memorial Hospital were obtained for RT-qPCR validation. All fresh samples were immediately snap-frozen in liquid nitrogen and stored at −80 °C until required. The use of tissues and clinical information in this study was approved by the Sun Yat-sen University’s Committees for Ethical Review of Research Involving Human Subjects (approval no. SYSEC-KY-KS-2020-201). All patients submitted their written informed consents. We state that we have complied with all relevant ethical regulations including the Declaration of Helsinki.

### Bioinformatics

Three pairs of different Gleason scored PCa tissues were sent for total RNA sequencing (RiboBio Co., Ltd. Guangzhou, China). In detail, total RNA was isolated from tissues using the Magzol Reagent (Magen, China) according to the manufacturer’s protocol, The quantity and integrity of RNA yield was assessed by using the K5500 (Beijing Kaiao, China) and the Agilent 2200 TapeStation (Agilent Technologies, USA) separately. Briefly, rRNAs were removed from Total RNA using QIAseq FastSelect-rRNA HRM KIT (QIAGEN, Germany) and fragmented to approximately 200 bp. Subsequently, the purified RNA fragments were subjected to first strand and then the second strand cDNA was synthesized using dUTP following by adaptor ligation and enrichment with a low-cycle according to instructions of NEBNext Ultra Directional RNA Library Prep Kit for Illumina (NEB, USA). The purified library products were evaluated using the Agilent 2200 TapeStation and Qubit (Thermo Fisher Scientific, USA). The libraries were sequenced by Illumina (Illumina, USA) with paired-end 150 bp at Ribobio Co. Ltd (Ribobio, China). EricScript software (version 0.5.5b) was applied using the hg38 reference genome and default parameters to analyze the RNA sequencing raw data and predict chimeric RNAs. We discarded chimeric RNAs with EricScore < 0.6. Blat filtering was applied to filter out false positive events followed by further filtering out events matching a list of chimeric RNAs from healthy individuals as described in our previous work^47^. RNA samples of *circCCDC7(15,16,17,18,19)* overexpressed PC3 and DU145 were also sent for polyA RNA sequencing to explore the possible downstream targets. GO term analysis (http://cbl-gorilla.cs.technion.ac.il/) was performed for the joint differential genes between PC3 and DU145 with hg38 background. The read counts of *circCCDC7(15,16,17,18,19)* in Chinese Prostate Cancer Genome and Epigenome Atlas (CPGEA) (http://www.cpgea.com/) were calculated by Agrep as our previous study described^48^. The Agrep read counts were normalized by the formula: normalized read counts = (read counts/ total reads)×10^9^.

### Cell culture

293 T (ATCC, CRL-3216), HCT116 (ATCC, CCL-247EMT), PrEC LH (ATCC, PCS-440-010), LNCaP (ATCC, CRL-1740), C4-2 (ATCC, CRL-3314), PC3 (ATCC, CRL-1435), DU145 (ATCC, HTB-81), NCI-H660 (ATCC, CRL-5813), and LASCPC-01 (ATCC, CRL-3356) were originally purchased from ATCC (American Type Culture Collection) and have been confirmed by STR genotyping in our previous studies^15,49–51^. We conduct routine testing for Mycoplasma every few months. PrEC LH, LNCaP, C4-2, and PC3 were cultured in RPMI 1640. 293 T, HCT116 and DU145 was cultured in Dulbecco’s modified Eagle’s medium (Gibco, USA), supplemented with 10% fetal bovine serum (Invitrogen, USA) and 1% pen/strep (Gibco, USA). NCI-H660 and LASCPC-01 were cultured in RPMI 1640 medium, supplemented with 0.005 mg/ml Insulin, 0.01 mg/ml Transferrin, 30 nM Sodium selenite, 10 nM Hydrocortisone, 10 nM beta-estradiol, 4 mM l-glutamine (HyCloneTM, USA) and 10% FBS. Cells were maintained at 5% CO_2_ in a 37 °C humidified incubator.

### RNA extraction, RNase treatment, ribosome extraction, and actinomycin D assay

Total RNA from cells was extracted using TRIzol (15596026, Thermo Fisher Scientific, USA) reagent as previously described^50^. Total RNA from clinical samples was harvested according to standard procedures as our previous study did^51^. In brief, 5 to 10 mg of tissue was added to a liquid nitrogen precooled mortar. The tissues were ground for 10 min, and 5 to 8 mL of liquid nitrogen was added every 1 min to keep the mortar cool. Then total RNA was isolated from the ground tissues by TRIzol.

cDNA was synthesized with random hexamer primer using Verso cDNA Synthesis Kit (AB-1453B, Thermo Fisher Scientific, USA). In brief, a 20 µL final reaction system was set up according to the table below. After incubation at 42 °C for 30 min, cDNA was obtained, followed by incubation at 95 °C for 2 min to inactivate the enzyme.

**Table:** 

	volume
5× cDNA synthesis buffer	4 μL
5 mM dNTP Mix	2 μL
400 ng/µL Random hexamers	1 μL
RT Enhancer	1 μL
Verso Enzyme Mix	1 μL
Template (RNA)	1–5 μL (1000 ng)
Water, nuclease-free	To 20 μL
Total volume	20 μL

RNase R (RNR07250, Lucigen, USA) treatment was used on RNAs extracted from tissues or cell lines at 37 °C for 30 min followed by 65 °C 20 min to enrich for circular RNAs.

Ribosome Extraction Kit (BB3606, BestBio, China) was applied to extract ribosomes from cultured cells. In brief, 1 × 10^7^ cells were collected and washed two times using PBS. Ribosomes were obtained according to the kit instructions, and then RNA was extracted from ribosomes using TRIzol as previously descried^52^.

The Nuclear and Cytoplasmic Extraction Kit (78833, Thermo Fisher Scientific, USA) was applied to isolate RNA from nuclear and cytoplasm fractions according to the manufacturer’s instructions. The Cytosolic and Membrane Extraction Kit (89842, Thermo Fisher Scientific, USA) was applied to isolate protein from cytosolic and membrane according to the manufacturer’s instructions. For actinomycin D assay, PC3 cells were treated with 2 mg/mL actinomycin D (11805017, Thermo Fisher Scientific, USA) to block transcription for 8, 16, and 24 h.

### RT-qPCR and touch-down PCR

Quantitative real-time PCR (RT-qPCR) was carried out on ABI StepOne Plus real time PCR system (Applied Biosystems, USA) using SYBR mix kit (AB-1285B, Thermo-Fisher Scientific, USA) as previously described^53^. In detail, a 20 µL final reaction was prepared up according to the table below. All primers used in this study were listed in Supplementary Table 4.

**Table:** 

	Volume
2× Absolute qPCR SYBR Green Capillary Mix	10 μL
10 μM Forward primer	0.4 μL
10 μM Reverse primer	0.4 μL
Template (cDNA)	2 μL
Water, nuclease-free	7.2 μL
Total volume	20 μL

The RT-qPCR reaction was performed according to the protocol below:

**Table:** 

Step	Temperature (°C)	Time	Number of cycles
Enzyme activation	95	15 min	1 cycle
Denaturation	95	15 s	40 cycles
Annealing	60	30 s	
Extension	72	45 s	

Touch-down PCR (TD-PCR) was carried out using Platinum Taq High Fidelity Kit (11304-011, Invitrogen, USA) to amplify the full length of *circCCDC7(15,16,17,18,19)* using divergent primers. In detail, a 50 µL final reaction was prepared according to the table below.

**Table:** 

	volume
10× High Fidelity PCR Buffer	5 μL
50 mM MgSO_4_	2 μL
10 mM dNTP Mix	1 μL
10 μM Forward Primer	1 μL
10 μM Reverse Primer	1 μL
cDNA	2 μL (<500 ng)
Platinum Taq DNA Polymerase High Fidelity (5 U/μL)	0.2 μL
Water, nuclease-free	To 50 μL

The TD-PCR reaction was performed according to the following protocol:

**Table:** 

Step	Temperature (°C)	Reaction time	
Initial denaturation	94 °C	2 min	
Denature	94 °C	30 s	Repeat ×2/×6/×10/×26 times from 58 °C to 52 °C
Anneal	58 °C	30 s	
Extension	68 °C	12 min	
Final extension	68 °C	10 min	
Hole	4 °C	indefinitely	

### Agarose electrophoresis and Sanger sequencing

2% Agarose Gel was used for separating PCR products. In detail, mix agarose (17850, Thermo Fisher Scientific, USA) powder with 1×TAE (Tris-base, Acetate and EDTA solution) in a microwavable flask. Microwave for 1–3 min to completely dissolve agarose followed by adding ethidium bromide (EtBr). Pour the agarose into a gel tray with the well comb in place and wait for 20–30 min until the gel completely solidified. PCR products were loaded into the wells of the gel and run at 120 V for 30 min. AxyPrep DNA Gel Extraction Kit (K210025, Thermo Fisher Scientific, USA) was used for gel extraction and DNA purification and followed by Sanger sequencing at Genewiz.

### Plasmid construction and transient transfection

The *circCCDC7(15,16,17,18,19)* and frame shift mutation fragments were cloned into the plenti-ciR-GFP-T2A vector (IGE Biotechnology, Guangzhou, China) to construct the overexpression plasmids. Before delivering the plasmids, the company conducted its own sequence validation. We have received the testing reports for all plasmids and confirmed all the sequences were correct. 293 T cells were used to package virus with the helper plasmids PMD2G and psPAX2. Stable cells that overexpress circular *circCCDC7(15,16,17,18,19)* were selected with puromycin. The linear *CCDC7* sequence was cloned into the pCDH-CMV-MCS-EF1-copGFP-T2A-Puro vector (IGE Biotechnology, Guangzhou, China) to construct the overexpression plasmid. For the transient transfection system, the plasmids above were transfected into 293 T, PC3 and DU145 with X-treme GENE HP DNA Transfection Reagent (6366546001, Roche, Basel, Switzerland) and cultured for 72 h for further investigation.

RNA interference (siRNA) oligonucleotides targeting *FLRT3* and negative control siRNAs were purchased from Thermo Fisher (s24376, s24377, and s24378). siRNA transfections were performed using 200 nM siRNA with 6 μL/mL Lipofectamine RNAimax (13778075, Thermo Fisher Scientific, USA) and incubated for 72 h for RNA isolation or protein collection.

### Protein isolation and Western blotting

Protein isolation and Western blotting were performed as described previously^53^. Ultracel-30 regenerated cellulose membrane (UFC8030, Sigma) was used to enrich the target circCCDC7-180aa from the cell culture supernatant. Primary antibodies: FLAG [1:1000; 14793; Cell Signaling Technology (CST)], FLRT3 (1:500; YN1973; ImmunoW), Slug (1:500; YN5478; ImmunoW), GAPDH (1:1000; 97166 S; CST), E-cadherin (1:500; YT1454; ImmunoW) and Claudin1 (1:1000; 13255; CST).

### Immunofluorescence, immunohistochemical scoring and ELISA

HCT116 cells were transfected with plenti-ciR *circCCDC7(15,16,17,18,19)*-GFP-T2A-Flag expressing plasmid for 72 h and seeded on chamber slides and subsequently co-fixed with 4% PFA and 0.5% Triton-X 100 for 10 min at 37 °C and blocked with 5% BSA in PBS. Cells were then incubated with primary antibody (FLAG) overnight at 4 °C, washed with PBS, and incubated with fluorescent secondary antibody for 1 h at 37 °C. Cells were then washed with PBS and incubated with DAPI for 10 min. Cells were subsequently examined under a Zeiss LSM 510 laser scanning fluorescence confocal microscope at 400× nominal magnification.

Immunohistochemical staining of FLRT3 from clinical samples were obtained from Proteinatlas (https://www.proteinatlas.org/index.php). We evaluated the immunostaining intensity of each sample as follows: negative = 0, weak = 1, moderate = 2, and strong = 3. We assessed the quantity of positively stained cells: negative = 0, <25% = 1, 25–75% = 2 and >75% = 3. The immunohistochemical score was calculated as the intensity score multiplied by the quantity score.

DYKDDDDK-Tag Detection ELISA Kit (501560, Cayman, British Overseas) was used to detect secreted protein encoded by *circCCDC7(15,16,17,18,19)* in extra-cellular environment according to the manufacturer’s instructions.

### Immunoprecipitation (IP) assays and Mass Spectrometry

Co-IP assays were performed according to the manufacturer’s instructions of the Pierce Crosslink Magnetic IP/Co-IP Kit (88805, Thermo Scientific) as our previous study described^54^. The proteins eluted from magnetic beads were sent to Wininnovate Bio (Shenzhen, China) for mass spectrometry analysis.

### Obtaining and concentration of the supernatant

Three days after 293 T transfected with vector or *circCCDC7(15,16,17,18,19)* plasmid, the supernatant was obtained and Ultra-4 Centrifugal Filter Unit (UFC803096-1, Merck millipore, USA) was used to enrich proteins in supernatant to 50 μl. The supernatant was then lyophilized by using freeze dryer (CV600, Jiaimu, Beijing).

### Cell proliferation, colony formation, migration, and invasion assays

For cell proliferation assay, cells (1000 for DU145 and 1500 for PC3 cells per well) were seeded in 96-well plates and cultured for 5 days. We measured the absorbance of each well at 450 nm every day using CCK8 (HY-K0301, MedChem Express, USA).

For the colony formation assay, cells (1000 for DU145 and 1500 for PC3 cells per well) were seeded in six-well plates and cultured in incubator for 7 days to form macroscopic clones. After staining with 0.1% crystal violet, we compared the difference among different groups.

The 24-well Transwell chamber (8 µm, 353097; Corning, USA) was used for the migration and invasion assay. In brief, 40,000 cells in 200 µL of 1% FBS medium were seeded in the top insert chamber, and 600 µL of medium containing 10% FBS was added into the lower chamber. The membranes in the top insert chamber were covered with Matrigel Basement Membrane Matrix (354234, Corning) for the cell invasion assay. The top chamber was fixed with 4% paraformaldehyde and stained with 0.2% crystal violet after incubation (12 h for DU145 and 48 h for PC3). The migrated cells on the lower membrane surface of the top chamber were counted under a microscope (Nikon, Tokyo, Japan).

### In vivo tumorigenesis experiments

All procedures involving animals were approved by the University of Virginia Institutional Animal Care and Use Committee. Immunocompromised BALB/c adult male mice (6–8 weeks old) were used. Animals were housed in sterilized plastic cages under specific pathogen-free conditions, at 22 °C, 12/12 light/dark cycle, 55% humidity. A total of 5 × 10^6^ DU145 cells were injected subcutaneously into both side of the dorsum (left side is control group, right side is *circCCDC7(15,16,17,18,19)* overexpressed group), and five mice were used here each time. At 6 weeks after implantation, the mice were euthanized by overdose Carbon dioxide (CO_2_), and the tumors were surgically dissected. The isolated tumor was weighed, and the volumes of the tumor were recorded using the following formula: tumor volume (mm^3^) = (length [mm]) × (width [mm])^2^ × 0.5.

### Statistical analyses

Clinical quantitative paired results from CPGEA and Sun Yat-sen memorial hospital in this study were assessed by Mann–Whitney test and Wilcoxon signed rank test (SPSS 20.0, Armonk, NY, USA). All other quantitative data are presented as the mean ± SD and were evaluated using GraphPad Prism 7.0. Statistical differences between the groups were assessed by one-way analysis of variance followed by Student’s *t*-test, and *p* ≤ 0.05 was considered significant. Spearman correlations were used to analyze the association of *circCCDC7(15,16,17,18,19)* with linear *CCDC7*. Fisher’s exact tests were used to analyze the association of *FLRT3* expression with clinicopathological characteristics by SPSS 22.0 software (SPSS Inc., Chicago, IL, USA). The Kaplan–Meier method was used to describe recurrence-free or relapse-free survival in patients from CPGEA or TCGA, and *p* ≤ 0.05 was considered statistically significant after Log-rank test.

### Reporting summary

Further information on research design is available in the Nature Research Reporting Summary linked to this article.

### Supplementary information

Supplementary Information

Reporting Summary

## Data Availability

The raw sequence data that PC3 and DU145 after *circCCDC7(15,16,17,18,19)* overexpressed have been deposited in the Genome Sequence Archive (Genomics, Proteomics & Bioinformatics 2021) in National Genomics Data Center (Nucleic Acids Res 2022), China National Center for Bioinformation / Beijing Institute of Genomics, Chinese Academy of Sciences (GSA-Human: HRA006338) that are publicly accessible at https://ngdc.cncb.ac.cn/gsa-human. The 3 pairs of clinical RNA-seq data from Sun Yat-sen memorial hospital was available from the corresponding author on reasonable request.
