# Supplementary material for: A protein-encoding CCDC7 circular RNA inhibits the progression of prostate cancer by up-regulating FLRT3
Source: NPJ Precis Oncol. 2024 Jan 16;8:11. doi: 10.1038/s41698-024-00503-2 (PMC10789799; doi:10.1038/s41698-024-00503-2)
Supplement: Supplementary file 1 — Supplementary Information [file 41698_2024_503_MOESM1_ESM.pdf]

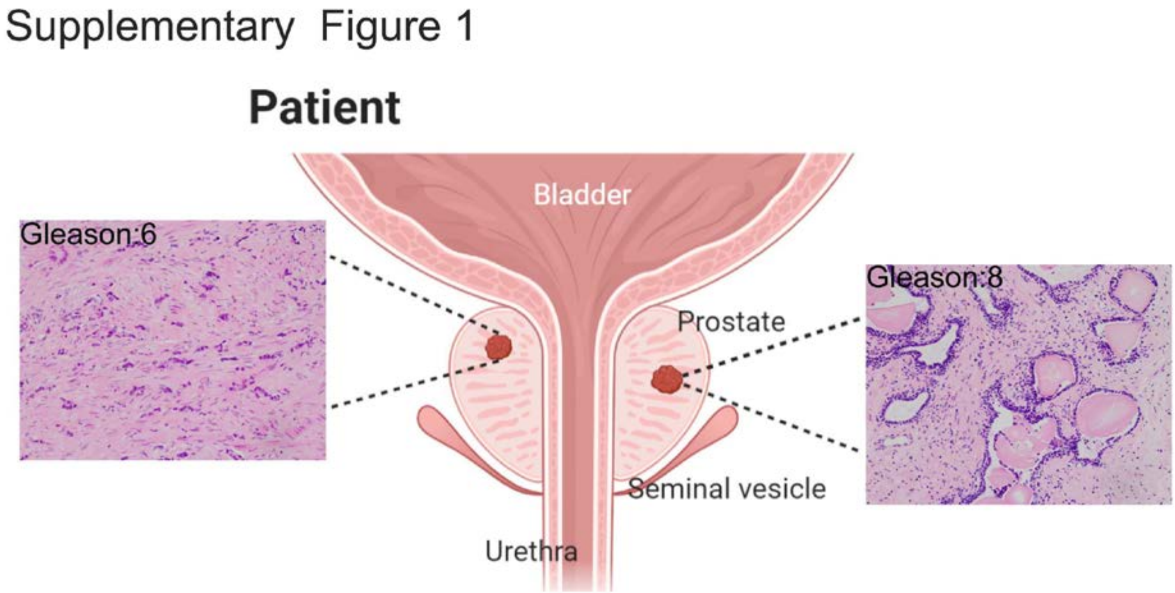

Supplementary Figure 1. Representative images of one of the PCa samples that met our selection criteria. The two tumor sites were distributed on two different lobes. Among those sites, left site: Gleason score = 3+3, right site: Gleason score = 4+4. In total, six samples were obtained from three patients.

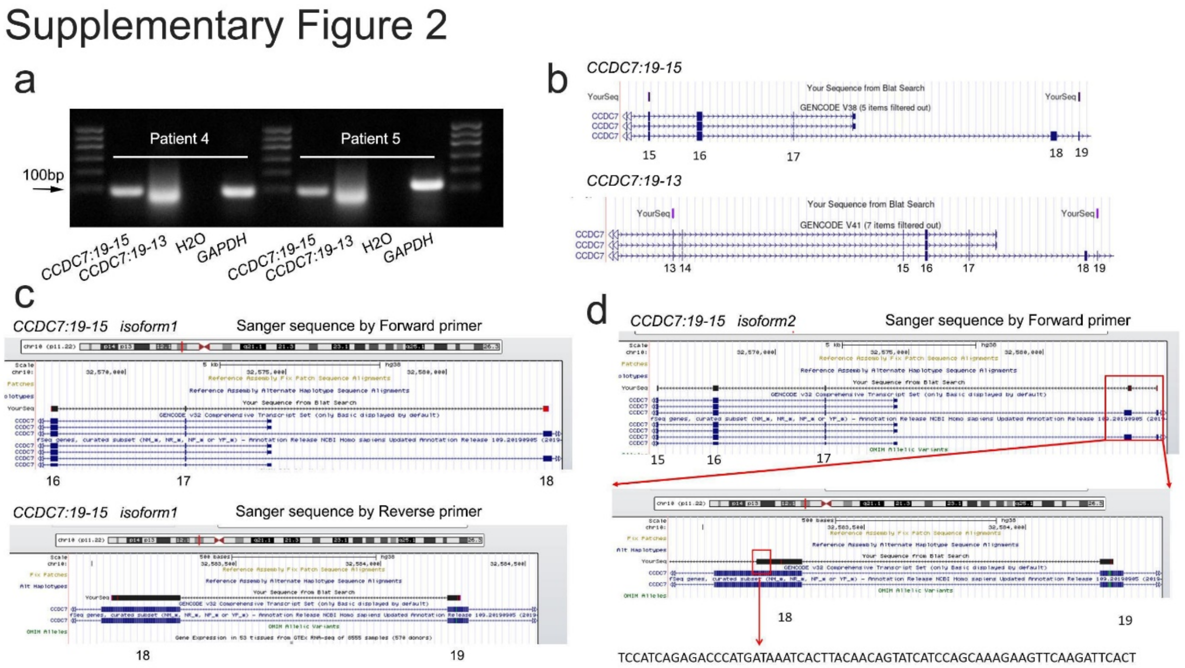

Supplementary Figure 2. Sanger sequencing result of *CCDC7*$_{19\text{-}15}$ and *CCDC7*$_{19\text{-}13}$, and full length of *circCCDC7(15,16,17,18,19)*. (a) Gel image of RT-qPCR product of *CCDC7*$_{19\text{-}15}$ and *CCDC7*$_{19\text{-}13}$ in another two clinical patients. Mix cDNA from tumor and adjacent normal margin was used. (b) Sanger sequence of the junction sites aligning to UCSC Genome Browser.

(c) Sanger sequence result of full length of *isoform1* aligning to UCSC Genome Browser. (d) Sanger sequencing result of full length *isoform2* aligning to UCSC Genome Browser.

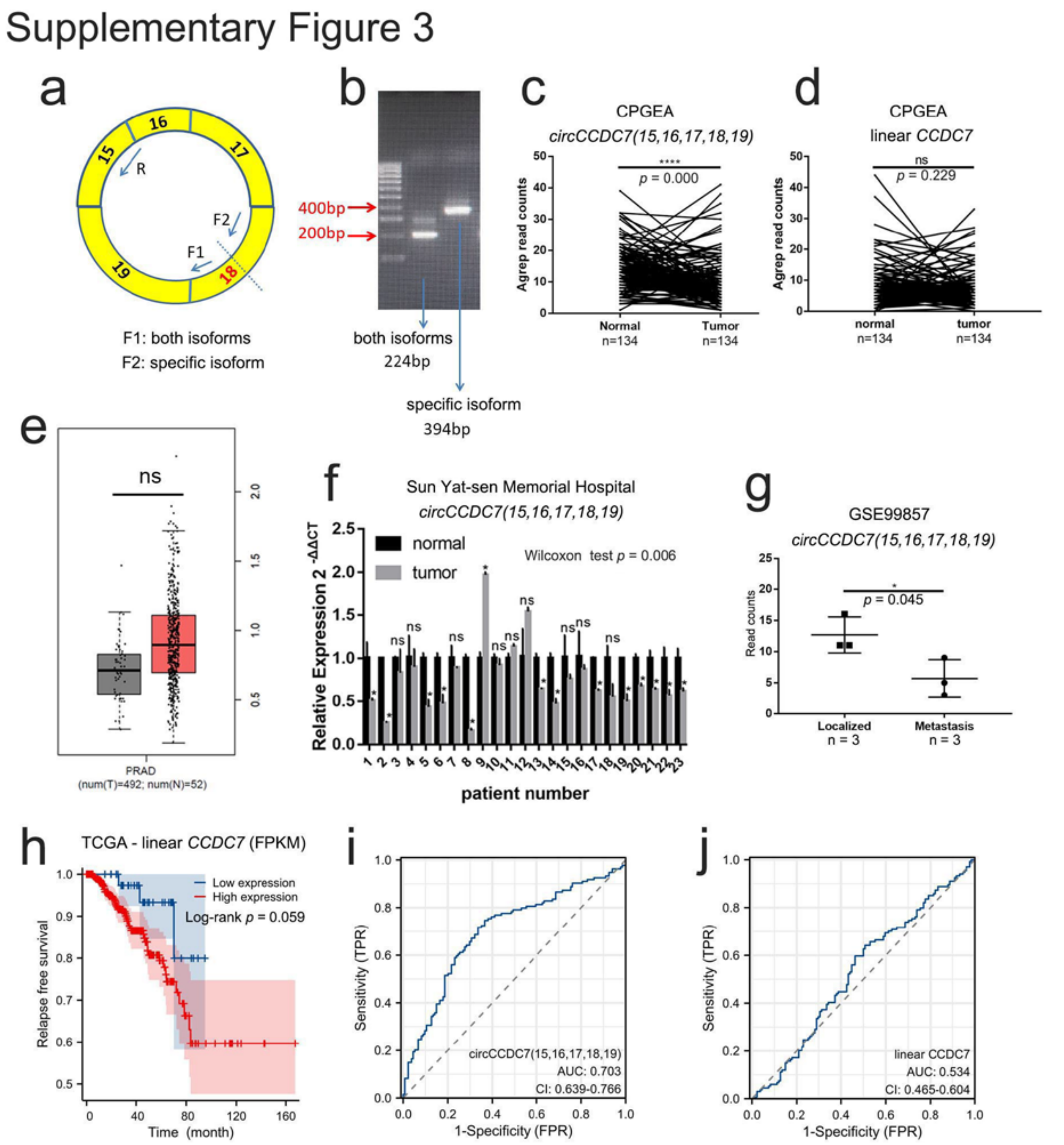

Supplementary Figure 3. Characterization of *circCCDC7(15,16,17,18,19)*. (a) Schematic diagram of *circCCDC7(15,16,17,18,19)* and specific primers design. (b) Gel image of RT-PCR product of two primers for both forms and specific forms. (c-d) Comparison of the expression of *circCCDC7(15,16,17,18,19)* (c) and linear *CCDC7* (d) in 134 pairs of PCa and normal margin samples from CPGEA using paired t test. (e) Expression of linear *CCDC7* from TCGA. (f) Quantitative analysis of *circCCDC7(15,16,17,18,19)* in 23 pairs of clinical samples

from Sun Yat-sen Memorial Hospital using paired t test. (g) Expression of *circCCDC7(15,16,17,18,19)* in metastasis dataset GSE99857 after AGREP analysis. (h) Relapse-free survival analysis of linear *CCDC7* using FPKM value TCGA. (i-j) AUC value of the ROC for normal or tumor by using the expression of *circCCDC7(15,16,17,18,19)* linear *CCDC7*. *$p < 0.05$, ****$p < 0.0001$.

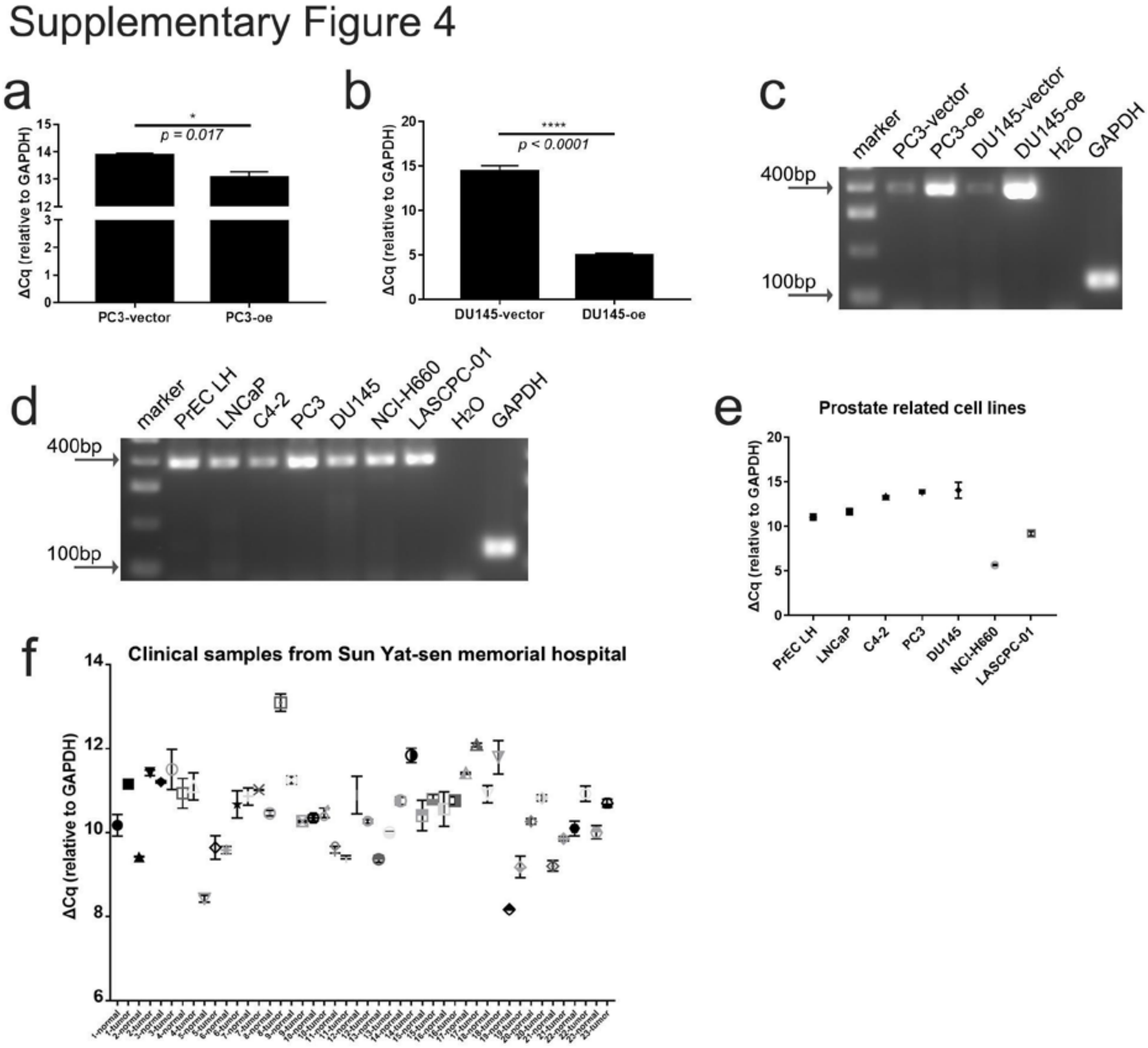

Supplementary Figure 4. The validation of PC3 and DU145 overexpression system. (a-b) The change of ΔCq in PC3 and DU145 after *circCCDC7(15,16,17,18,19)* overexpressed. (c) Gel image of RT-qPCR product of *circCCDC7(15,16,17,18,19)* when it was stable overexpressed in PC3 and DU145. (d) Gel image of RT-qPCR product of *circCCDC7(15,16,17,18,19)* in current wide-type prostate related cell lines. (e) The ΔCq value of *circCCDC7(15,16,17,18,19)* in current wide-type prostate related cell lines. (f) The ΔCq

value of *circCCDC7(15,16,17,18,19)* in 23 pairs of clinical samples from Sun Yat-sen memorial hospital. *$p < 0.05$, ****$p < 0.0001$.

Supplementary Figure 5

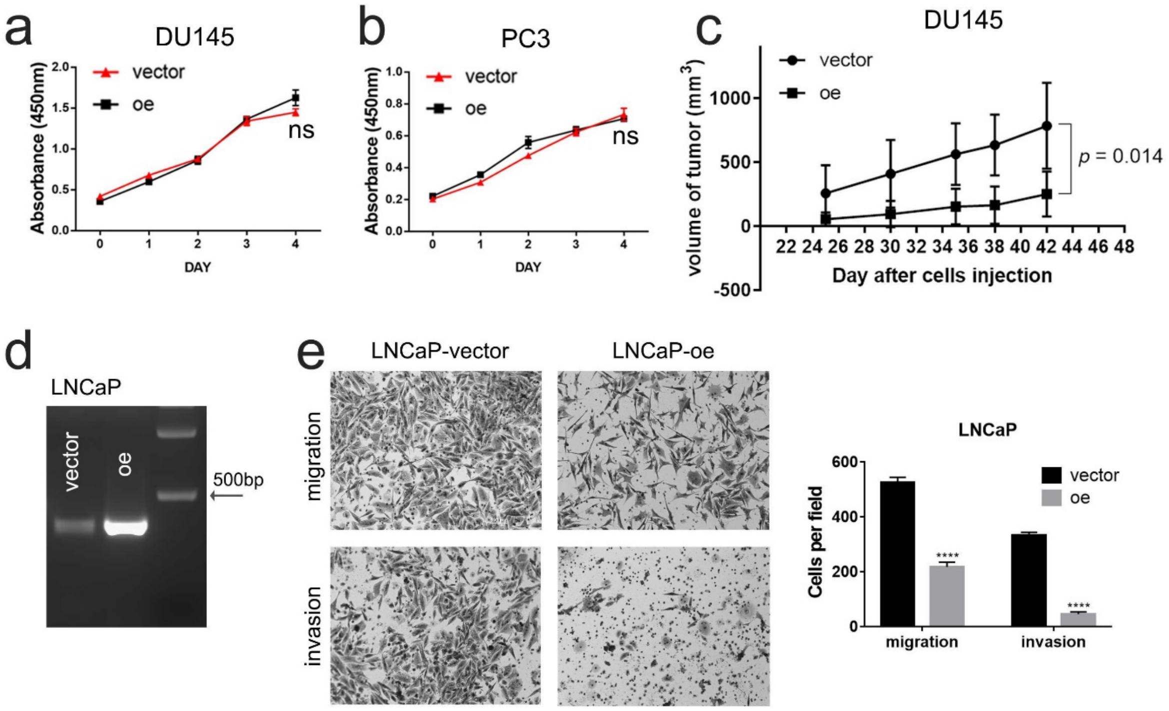

Supplementary Figure 5. *CircCCDC7(15,16,17,18,19)* inhibits the viability, migration, and invasion of PCa. (a-b) CCK8 assay measuring cell proliferation in DU145 and PC3 cell lines transfected with *circCCDC7(15,16,17,18,19)* overexpression plasmid or control vector. (c) Tumor volume increase curves of the *circCCDC7(15,16,17,18,19)* overexpression and control groups are summarized in the line chart after subcutaneous injection. (d) Gel image of RT-qPCR product of *circCCDC7(15,16,17,18,19)* when LNCap was transfected with *circCCDC7(15,16,17,18,19)* plasmid. (e) Representative images of migration and invasion assays using LNCaP after different plasmids transfection, and the histogram analysis of migrated and invaded cell counts.

Supplementary Figure 6

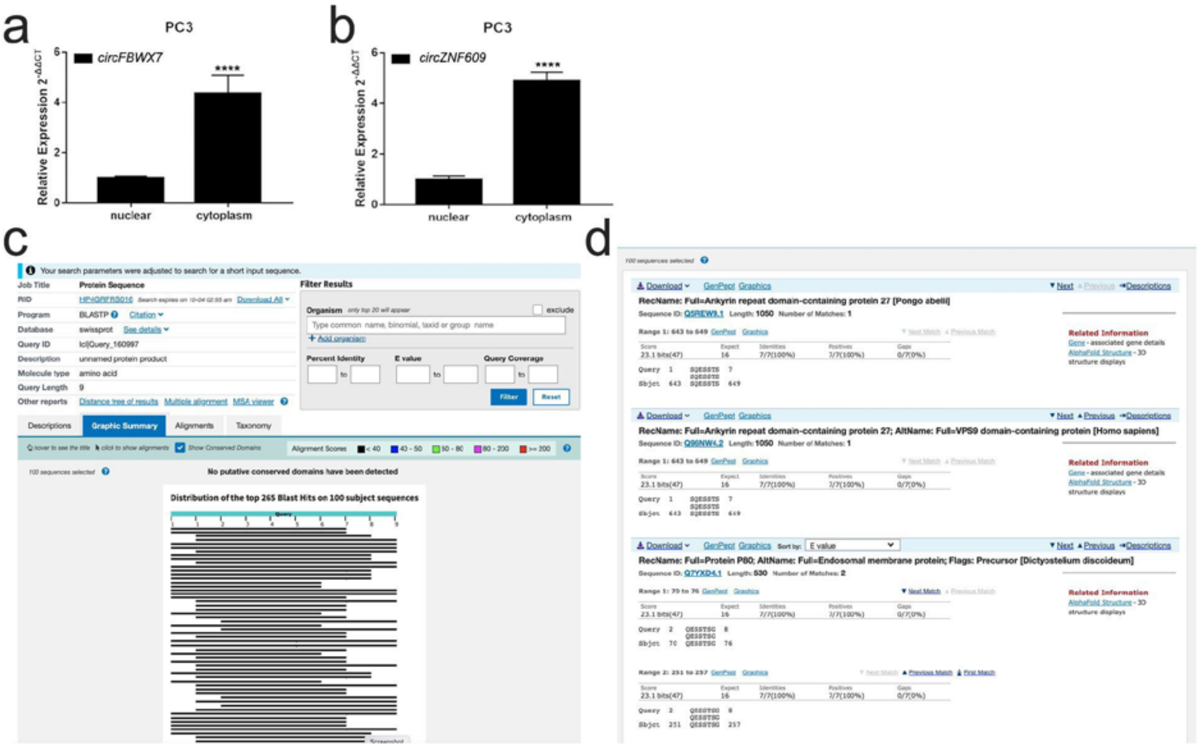

Supplementary Figure 6. CircCCDC7-180aa cannot be derived from a linear transcript of another gene. (a-b) Quantitative analysis on the expression of *circFBXW7* and *circZNF609* between nuclear and cytoplasm in PC3 cells. (c-d) The specific junction amino acids (SQESSTSGN) was used to BLASTP against UniProtKB/Swiss-Prot database (https://www.uniprot.org) and found no exact match.

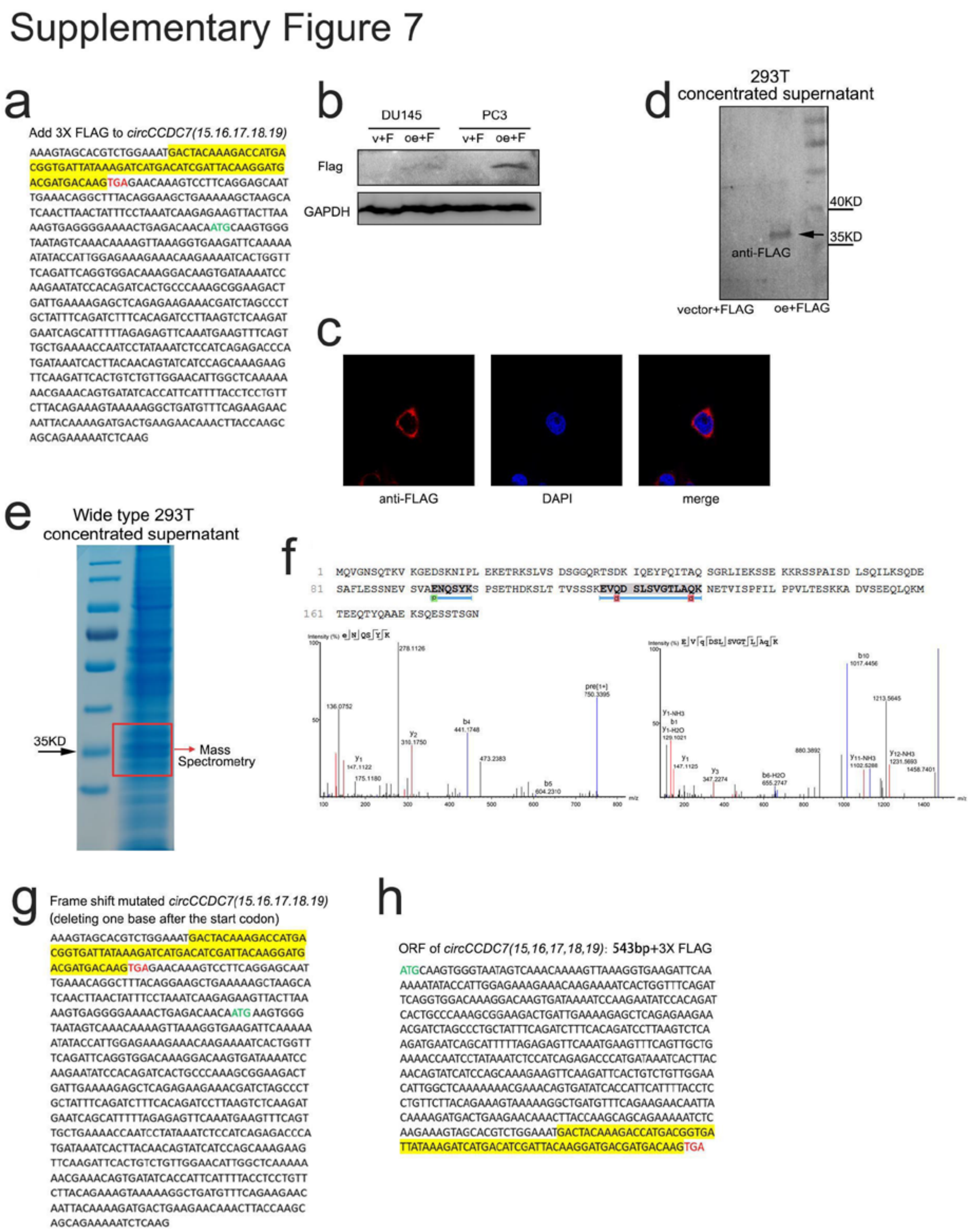

Supplementary Figure 7. ***CircCCDC7(15,16,17,18,19)*** **encodes a protein which can be secreted.** (a) 3× FLAG was added just before the stop codon (yellow highlights). (b) FLAG tag antibody was used to detect circCCDC7-180aa expression in PC3 and DU145 cells after transfection. (c) Immunofluorescence assay demonstrated that circCCDC7-180aa was mainly located around nucleus in du145 cell line which *circCCDC7(15,16,17,18,19)* was stable

overexpressed. (d) The supernatant from 293T cells was lyophilized, following by western blot to measure the secreted protein. (e-f) The supernatant from 293T cells was used to do IP and mass spectrometry, which identified the peptides of circCCDC7-180aa. (g-h) The full length of frame shift mutation plasmid and linearized *circCCDC7(15,16,17,18,19)* plasmid.

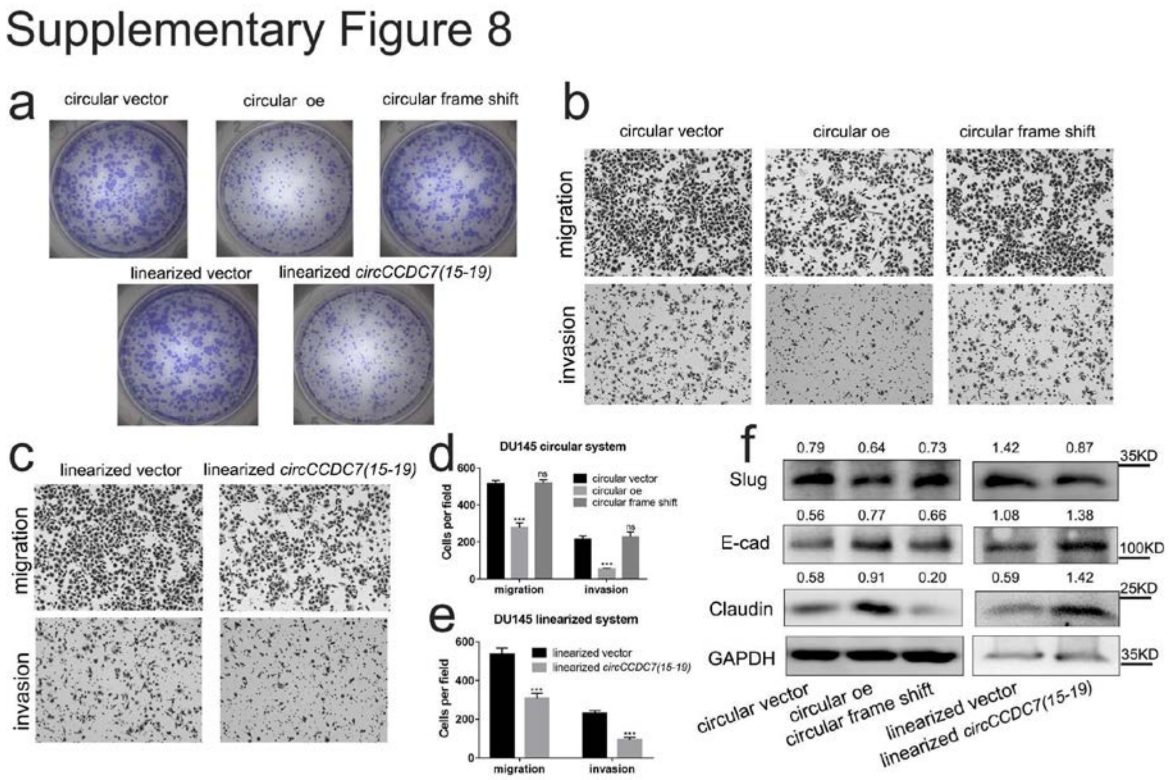

Supplementary Figure 8. *CircCCDC7(15,16,17,18,19)* suppresses the cell viability, migration, and invasion of DU145 by its encoded protein. (a) Colony formation assay measuring cell viability in DU145 cells when different plasmids were transfected. (b-c) Representative images of migration and invasion assays using DU145 after different plasmids transfection. (d-e) Histogram analysis of migrated and invaded cell counts. (f) Representative image of the western blotting analysis of Slug, E-cad, and Claudin protein levels after different plasmids transfection. ***p < 0.001.

Supplementary Figure 9

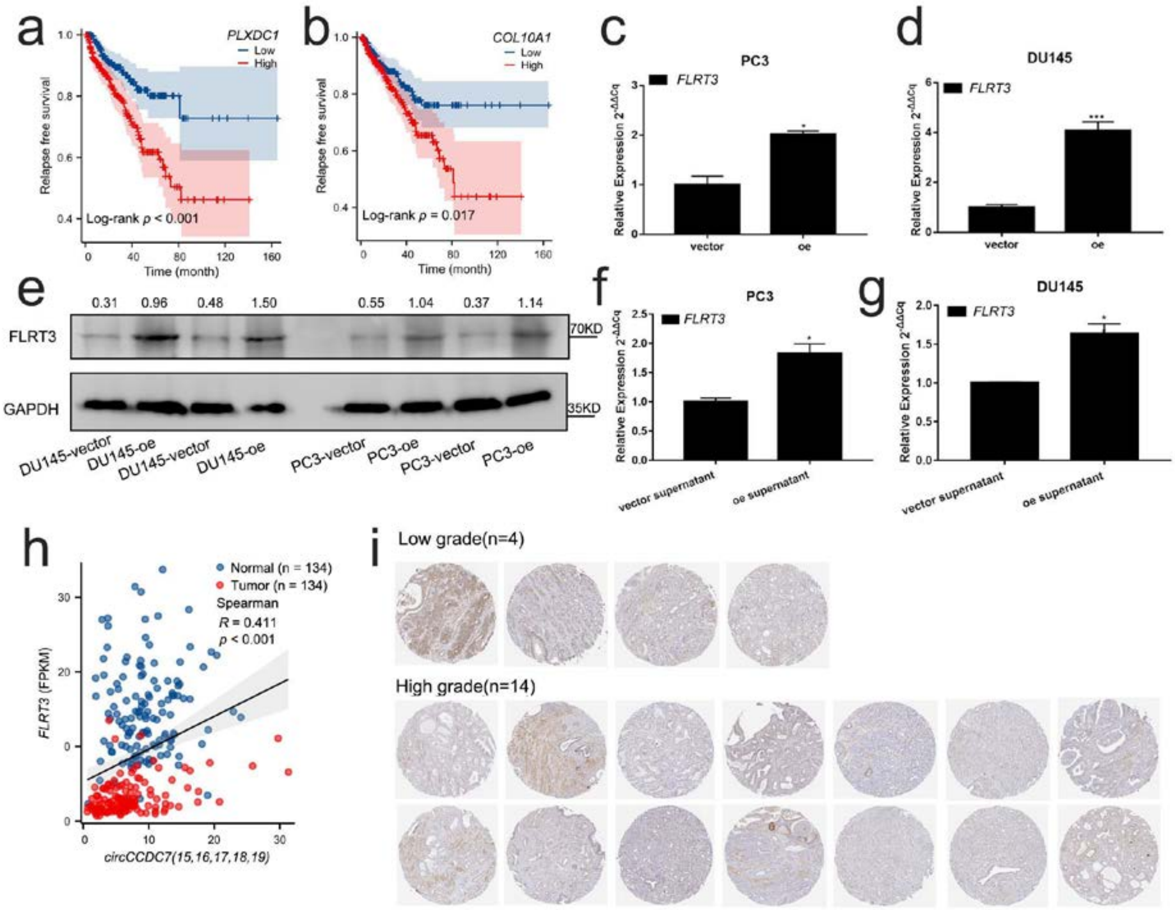

Supplementary Figure 9. ***FLRT3* is a potential downstream of *circCCDC7(15,16,17,18,19)*.** (a-b) Relapse-free survival analysis of PLXDC1 (a) and COL10A1 (b) in TCGA. (c-d) Quantitative analysis expression of *FLRT3* in PC3 and DU145 when *circCCDC7(15,16,17,18,19)* was overexpressed. (e) Representative image of the western blotting analysis of FLRT3 protein levels when *circCCDC7(15,16,17,18,19)* was overexpressed in DU145 and PC3. The grayscale ratio of FLRT3 to GAPDH was listed on top of the lanes. (f-g) Quantitative analysis expression of *FLRT3* in wide-type PC3 and DU145 when treated with supernatant from *circCCDC7(15,16,17,18,19)* overexpressed 293T cells. (h) The correlation between *circCCDC7(15,16,17,18,19)* and *FLRT3* in CPGEA dataset. (i) Photomicrographs of the immunohistochemical staining of FLRT3 in the Proteinatlas. *p < 0.05, ***p < 0.001.

Supplementary Figure 10

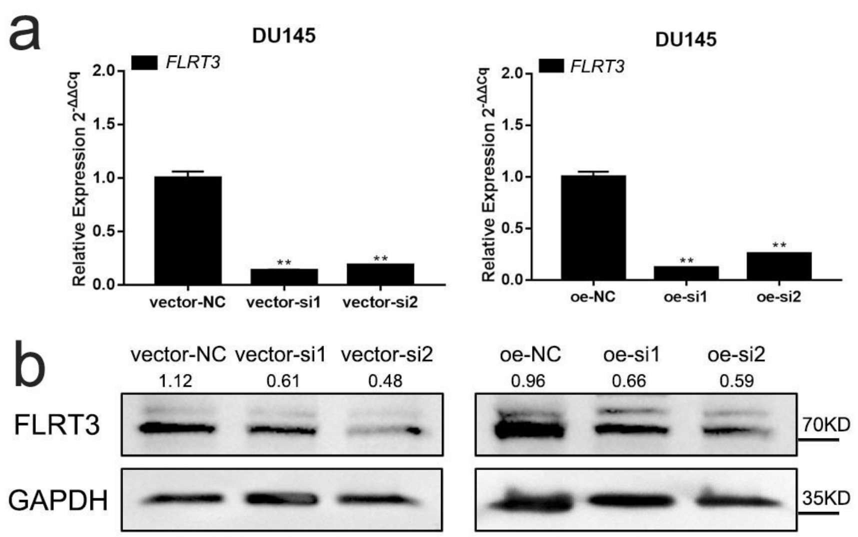

Supplementary Figure 10. RT-qPCR (a) and western blot (b) analysis of FLRT3 expression levels in *FLRT3*-knockdown cells with *circCCDC7(15,16,17,18,19)* overexpressing combined with *FLRT3* knocking down. The grayscale ratio of FLRT3 to GAPDH was listed on top of the lanes. **p < 0.01.

Supplementary Figure 11

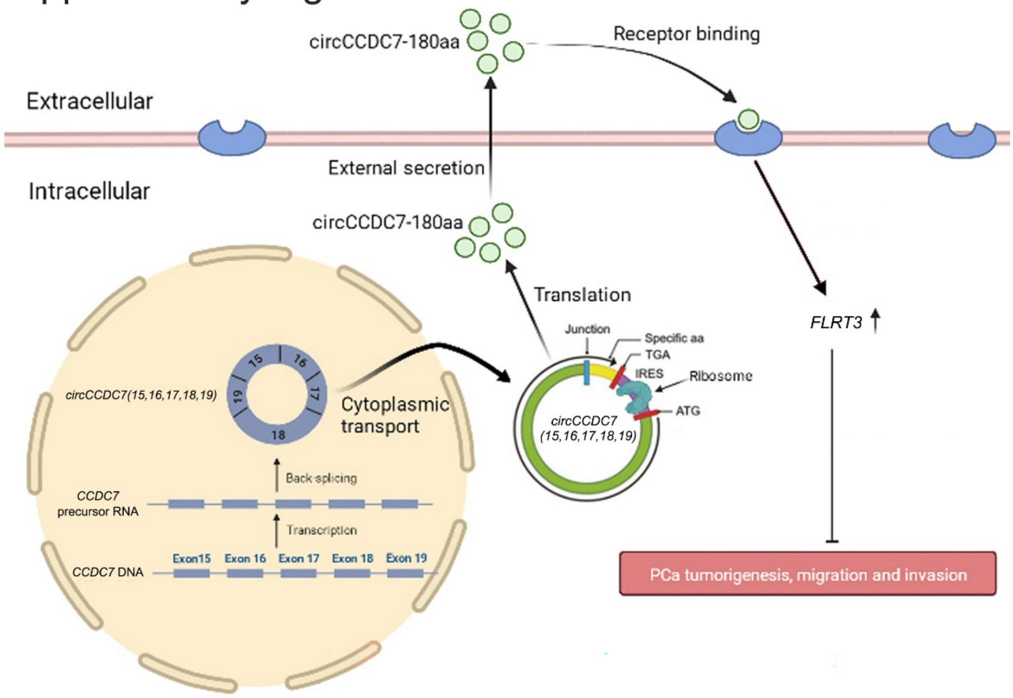

Supplementary Figure 11. Schematic diagram of *circCCDC7(15,16,17,18,19)* influencing prostate cancer.

Supplementary Table 1. Prognostic value of *circCCDC7(15,16,17,18,19)* expression for the survival by Cox proportional hazards model.

**Variable**	**HR (95% CI)**	***p***
**Univariable analysis**		
Age (≥70 vs. < 70)	1.404 (0.717-2.751)	0.323
Gleason score (<7 vs.≥7)	0.039 (0.000-3.856)	0.166
Tumor stage (T2 vs. T3–T4)	0.561 (0.282-1.114)	0.099
Lymph node stage (N0 vs. N1)	0.369 (0.166-0.820)	0.014*
PSA (<20 vs. ≥20)	0.250 (0.113-0.552)	0.001***
Bone metastasis (Yes vs. No)	0.335 (0.117-0.957)	0.041*
Distant metastasis (M0 vs. M1)	0.553 (0.192-1.597)	0.274
Seminal Vesical (Yes vs. No)	0.338 (0.164-0.695)	0.003**
Surgical Margin (Yes vs. No)	0.540 (0.275-1.062)	0.074
circCCDC7 (Low vs. High)	2.612 (1.070-6.378)	0.035*
**Multivariable analysis**		
Gleason score (<7 vs.≥7)	0.000 (0.000-)	0.977
Lymph node stage (N0 vs. N1)	0.500 (0.211-1.187)	0.116
PSA (<20 vs. ≥20)	0.445 (0.186-1.063)	0.068
Bone metastasis (Yes vs. No)	0.341 (0.106-1.091)	0.070
Seminal Vesical (Yes vs. No)	0.433 (0.192-0.979)	0.044*
circCCDC7 (Low vs. High)	2.855 (1.132-7.354)	0.026*

Cut off value of *circCCDC7(15,16,17,18,19)* score: 10% low expression *vs* 90% high expression; PSA, prostate-specific antigen; T, tumor; N, node; M, metastasis. *$p \leq 0.05$, **$p \leq 0.01$, ***$p \leq 0.001$.

Note: Univariable Cox regression using the log transformed *circCCDC7(15,16,17,18,19)* did not result in a significant result.

Supplementary Table 2. Correlation of *FLRT3* expression with clinico-pathologic characteristics of PCa patients in TCGA database.

Clinical features	Total patients, *n*	Low, *n* (%)	High, *n* (%)	*P*
**Age, years**				0.745
≤65	354	175 (49.4%)	179(50.6%)	
>65	143	73 (51%)	70 (49%)	
**Gleason score**				0.012*
≤7	292	132 (45.2%)	160 (54.8%)	
＞7	205	116 (56.6%)	89 (43.4%)	
**Serum PSA levels, ng/ml**				0.13
≤4	413	198 (47.9%)	215 (52.1%)	
>4	27	17 (63%)	10 (37%)	
**T stage**				0.364
T1-T2	187	89 (47.6%)	98 (52.4%)	
T3-T4	303	157 (51.8%)	146 (48.2%)	
**Lymph node metastasis**				0.178
N0	346	168 (48.6%)	178 (51.4%)	
N1	79	45 (57%)	34 (43%)	
**Distant metastasis**				0.258
M0	454	229 (50.4%)	225 (49.6%)	
M1	3	3 (100%)	0 (0%)	

Cut off value of *FLRT3* score: medium; PSA, prostate-specific antigen; T, tumor; N, node; M, metastasis. *$P < 0.05$.

Supplementary Table 3. Calculation of IHC score of FLRT3 in Proteinatlas.

Patient	Grade	intensity	Quantity	Score
1	Low	2	3	6
2	Low	2	3	6
3	Low	2	2	4
4	Low	2	2	4
5	High	1	3	3
6	High	1	1	1
7	High	1	2	2
8	High	2	3	6
9	High	0	0	0
10	High	1	1	1
11	High	1	2	2
12	High	1	3	3
13	High	1	2	2
14	High	2	3	6
15	High	0	0	0
16	High	1	1	1
17	High	2	2	4
18	High	2	2	4

Supplementary Table 4. The sequences of primers used in this study.

NO.	Target	Forward (5'-3')	Reverse (5'-3')
**1**	**Fusion *CCDC7: 19-15***	ACAAACTTACCAAGCAGCAG	AGCCTGTTTCAATTGCTCCT
**2**	**Fusion *CCDC7: 19-13***	ACAAACTTACCAAGCAGCAG	CACAGCTTTCTTTGTTCGAT
**3**	**Linear *CCDC7***	TCCGTACAACGTCAAGAAGG	AAAGCTTGGACCAGCCCTAT
**4**	**Divergent *CCDC7: 19-15***	AATCTCAAGAAAGTAGCACGTC	CTGCTGCTTGGTAAGTTTGTT
**5**	**All isoforms of *circCCDC7(15,16,17,18,19)***	CAAGATTCACTGTCTGTTGG	AGCCTGTTTCAATTGCTCCT
**6**	**Specific isoform of *circCCDC7(15,16,17,18,19)***	AACGATCTAGCCCTGCTATT	AGCCTGTTTCAATTGCTCCT
**7**	***circFBWX7***	CAGTGTCACGAACTCCAGTA	CACATTCCTCTGACCCAGTA
**8**	***circITCH***	AAGGAGCAATGCAGCAGTTT	AGTCACAACTACTTCTTCAACCCAT
**9**	***FLRT3***	ACAGTGTATCCTGCCCAAGG	AGCCAGCTGAGTCTCAAAGC
**10**	***GAPDH***	CAAGGCTGAGAACGGGAAG	TGAAGACGCCAGTGGACTC
