# Supplementary material for: A protein-encoding CCDC7 circular RNA inhibits the progression of prostate cancer by up-regulating FLRT3
Source: NPJ Precis Oncol. 2024 Jan 16;8:11. doi: 10.1038/s41698-024-00503-2 (PMC10789799; doi:10.1038/s41698-024-00503-2)
Supplement: Supplementary file 2 — Reporting Summary [file 41698_2024_503_MOESM2_ESM.pdf]

nature portfolio

Corresponding author(s): Hui Li and Hai Huang

Last updated by author(s): Dec 23, 2023

Reporting Summary

Nature Portfolio wishes to improve the reproducibility of the work that we publish. This form provides structure for consistency and transparency in reporting. For further information on Nature Portfolio policies, see our Editorial Policies and the Editorial Policy Checklist.

Statistics

For all statistical analyses, confirm that the following items are present in the figure legend, table legend, main text, or Methods section.

n/a	Confirmed	
☐	☒	The exact sample size (*n*) for each experimental group/condition, given as a discrete number and unit of measurement
☐	☒	A statement on whether measurements were taken from distinct samples or whether the same sample was measured repeatedly
☐	☒	The statistical test(s) used AND whether they are one- or two-sided<br>*Only common tests should be described solely by name; describe more complex techniques in the Methods section.*
☒	☐	A description of all covariates tested
☒	☐	A description of any assumptions or corrections, such as tests of normality and adjustment for multiple comparisons
☐	☒	A full description of the statistical parameters including central tendency (e.g. means) or other basic estimates (e.g. regression coefficient) AND variation (e.g. standard deviation) or associated estimates of uncertainty (e.g. confidence intervals)
☐	☒	For null hypothesis testing, the test statistic (e.g. *F*, *t*, *r*) with confidence intervals, effect sizes, degrees of freedom and *P* value noted<br>*Give P values as exact values whenever suitable.*
☒	☐	For Bayesian analysis, information on the choice of priors and Markov chain Monte Carlo settings
☒	☐	For hierarchical and complex designs, identification of the appropriate level for tests and full reporting of outcomes
☐	☒	Estimates of effect sizes (e.g. Cohen's *d*, Pearson's *r*), indicating how they were calculated

Our web collection on statistics for biologists contains articles on many of the points above.

Software and code

Policy information about availability of computer code

Data collection	Some data used in this study was download from the open source. TCGA: https://portal.gdc.cancer.gov/. CPGEA: http://www.cpgea.com/.
Data analysis	State that no software was used

For manuscripts utilizing custom algorithms or software that are central to the research but not yet described in published literature, software must be made available to editors and reviewers. We strongly encourage code deposition in a community repository (e.g. GitHub). See the Nature Portfolio guidelines for submitting code & software for further information.

Data

Policy information about availability of data

All manuscripts must include a data availability statement. This statement should provide the following information, where applicable:

- Accession codes, unique identifiers, or web links for publicly available datasets
- A description of any restrictions on data availability
- For clinical datasets or third party data, please ensure that the statement adheres to our policy

We have no data availability statement here.

Research involving human participants, their data, or biological material

Policy information about studies with human participants or human data. See also policy information about sex, gender (identity/presentation), and sexual orientation and race, ethnicity and racism.

Reporting on sex and gender	This study was lack of sex- and gender-based analysis because the prostate cancer can only be found in male.
Reporting on race, ethnicity, or other socially relevant groupings	This study didn't describe people according to race, ethnicity or other socially constructed categories.
Population characteristics	There was no covariate population characteristic of the human paticipants
Recruitment	The tissues and clinical information was used with written informed consents from the patients who have undergone radical prostatectomy from 2020 to 2021.
Ethics oversight	Sun Yat-sen University's Committees for Ethical Review of Research Involving Human Subjects

Note that full information on the approval of the study protocol must also be provided in the manuscript.

Field-specific reporting

Please select the one below that is the best fit for your research. If you are not sure, read the appropriate sections before making your selection.

☒ Life sciences ☐ Behavioural & social sciences ☐ Ecological, evolutionary & environmental sciences

For a reference copy of the document with all sections, see nature.com/documents/nr-reporting-summary-flat.pdf

Life sciences study design

All studies must disclose on these points even when the disclosure is negative.

Sample size	23 pairs of clinical samples from cancer and adjacent areas were used. The larger the sample size, the more it can get the right conclusion. It is generally believed that clinical data exceeding 5 pairs can get a conclusion, including many single cell sequencing and RNA sequencing studies.
Data exclusions	No data was excluded from the analysis
Replication	All attempts at replication were successful.
Randomization	All the samples were allocated as paired tumor and normal from the same patient.
Blinding	Blinding was not relevant to this study because we need to distinguish tumor samples from adjacent normal.

Reporting for specific materials, systems and methods

We require information from authors about some types of materials, experimental systems and methods used in many studies. Here, indicate whether each material, system or method listed is relevant to your study. If you are not sure if a list item applies to your research, read the appropriate section before selecting a response.

Materials & experimental systems

n/a	Involved in the study	
☐	☒	Antibodies
☐	☒	Eukaryotic cell lines
☒	☐	Palaeontology and archaeology
☐	☒	Animals and other organisms
☐	☒	Clinical data
☒	☐	Dual use research of concern
☒	☐	Plants

Methods

n/a	Involved in the study	
☒	☐	ChIP-seq
☒	☐	Flow cytometry
☒	☐	MRI-based neuroimaging

Antibodies

Antibodies used	Primary antibodies: FLAG [1:1000; 14793; Cell Signaling Technology (CST)], FLRT3 (1:500; YN1973; ImmunoW), Slug (1:500; YN5478; ImmunoW), GAPDH (1:1000; 97166S; CST), E-cadherin (1:500; YT1454; ImmunoW) and Claudin1 (1:1000; 13255; CST).

Validation	The validation of all the primary antibodies above were come from the companies' website: http://www.immunoway.com/ and https://www.cellsignal.cn/. The company has provided corresponding verification documents on their website.

Eukaryotic cell lines

Policy information about cell lines and Sex and Gender in Research

Cell line source(s)	293T, HCT116, PrEC LH, LNCaP, C4-2, PC3, DU145, NCI-H660, and LASCPC-01 were purchased from ATCC (American Type Culture Collection).
Authentication	The cell lines above were originally purchased from ATCC and have been confirmed by STR genotyping in our previous studies.
Mycoplasma contamination	We conduct routine testing for Mycoplasma every few months and the results showed no Mycoplasma contamination.
Commonly misidentified lines (See ICLAC register)	Not applicable

Animals and other research organisms

Policy information about studies involving animals; ARRIVE guidelines recommended for reporting animal research, and Sex and Gender in Research

Laboratory animals	Immunocompromised BALB/c adult male mice
Wild animals	Immunocompromised BALB/c adult male mice (6–8 weeks old)
Reporting on sex	Findings only apply to one sex because the prostate cancer can only be found in male.
Field-collected samples	Animals were housed in sterilized plastic cages under specific pathogen-free conditions, at 22°C, 12/12 light/dark cycle, 55% humidity. The end of the experiments was the time when the tumor volume exceed 1500mm3.
Ethics oversight	University of Virginia Institutional Animal Care and Use Committee

Note that full information on the approval of the study protocol must also be provided in the manuscript.

Clinical data

Policy information about clinical studies

All manuscripts should comply with the ICMJE guidelines for publication of clinical research and a completed CONSORT checklist must be included with all submissions.

Clinical trial registration	Not applicable
Study protocol	Not applicable
Data collection	Not applicable
Outcomes	Not applicable

Plants

Seed stocks	Not applicable
Novel plant genotypes	Not applicable
Authentication	Not applicable
